# Important Role of FTO in the Survival of Rare Panresistant Triple-Negative Inflammatory Breast Cancer Cells Facing a Severe Metabolic Challenge

**DOI:** 10.1371/journal.pone.0159072

**Published:** 2016-07-08

**Authors:** Balraj Singh, Hannah E. Kinne, Ryan D. Milligan, Laura J. Washburn, Mark Olsen, Anthony Lucci

**Affiliations:** 1 Department of Breast Surgical Oncology, and Morgan Welch Inflammatory Breast Cancer Research Program and Clinic, The University of Texas MD Anderson Cancer Center, Houston, Texas, United States of America; 2 Department of Pharmaceutical Sciences, College of Pharmacy–Glendale, Midwestern University, Glendale, Arizona, United States of America; University of South Alabama, UNITED STATES

## Abstract

We have previously shown that only 0.01% cells survive a metabolic challenge involving lack of glutamine in culture medium of SUM149 triple-negative Inflammatory Breast Cancer cell line. These cells, designated as SUM149-MA for metabolic adaptability, are resistant to chemotherapeutic drugs, and they efficiently metastasize to multiple organs in nude mice. We hypothesized that obesity-related molecular networks, which normally help in cellular and organismal survival under metabolic challenges, may help in the survival of MA cells. The fat mass and obesity-associated protein FTO is overexpressed in MA cells. Obesity-associated cis-acting elements in non-coding region of *FTO* regulate the expression of *IRX3* gene, thus activating obesity networks. Here we found that IRX3 protein is significantly overexpressed in MA cells (5 to 6-fold) as compared to the parental SUM149 cell line, supporting our hypothesis. We also obtained evidence that additional key regulators of energy balance such as ARID5B, IRX5, and CUX1 P200 repressor could potentially help progenitor-like TNBC cells survive in glutamine-free medium. MO-I-500, a pharmacological inhibitor of FTO, significantly (>90%) inhibited survival and/or colony formation of SUM149-MA cells as compared to untreated cells or those treated with a control compound MO-I-100. Curiously, MO-I-500 treatment also led to decreased levels of FTO and IRX3 proteins in the SUM149 cells initially surviving in glutamine-free medium as compared to MO-I-100 treatment. Interestingly, MO-I-500 treatment had a relatively little effect on cell growth of either the SUM149 or SUM149-MA cell line when added to a complete medium containing glutamine that does not pose a metabolic challenge. Importantly, once selected and cultured in glutamine-free medium, SUM149-MA cells were no longer affected by MO-I-500 even in Gln-free medium. We conclude that panresistant MA cells contain interconnected molecular networks that govern developmental status and energy balance, and genetic and epigenetic alterations that are selected during cancer evolution.

## Introduction

Cancer resembles an evolution-like process in the body, involving epigenetic and genetic alterations in tumor cells accompanied by a selection process that eliminates a majority of cancer cells [1–3]. Immune surveillance is one of the multiple challenges that cancer cells would face before metastasis as they try to colonize at a distant organ site. It is well accepted at this time that only a small percentage of cancer cells present in the overall population may have an ability to generate an embryo-like cellular heterogeneity that may provide a survival advantage during cancer evolution. Despite tremendous technical advances leading to our present understanding of the genomic landscape of cancer, our ability to overcome therapeutic resistance in advanced cancers remains limited. Our ability to improve treatment of advanced cancers that do not respond to currently offered therapies will depend largely on how well we do against the rare but highly adaptable cells that drive cancer evolution and therapy resistance. It is commonly accepted that we must find ways to apply effective combination therapies in a timely manner to improve outcomes of patient survival, but there are serious hurdles in identifying and implementing such therapies. To facilitate this task, we have developed a usable *in vitro* system of realistic intrinsic resistance in highly heterogeneous triple negative breast cancer (TNBC) [4–6] for testing new combination therapies [7, 8].

It is difficult to model the intrinsic resistance that may be imparted by rare but adaptable, possibly panresistant, cancer cells into usable systems for evaluating therapies. Most therapies are developed based on studies with the cell lines that have been established from primary tumors or metastases. Although it is likely that cancer cells are heterogeneous with regard to their metabolic state, it is not an easy task to optimize cultures of specific subpopulations of cancer cells [9]. In fact, this may be one of the weakest links in the drug development process. To stress this point, even if one establishes a cell line from a metastatic legion, many subpopulations of cancer cells that impart intrinsic resistance are lost because *in vitro* culture conditions are not suitable for their survival and growth. It is likely that most cells that proliferate in the artificial culture conditions lose their capabilities to survive, grow, and metastasize in the body. As a possible manifestation of this limitation, one has to inject a large number of cancer cells as xenografts into immunocompromised mice to get tumor growth; only some cell lines produce tumors that can metastasize in these models.

Most drugs are evaluated for their ability to inhibit cell proliferation and/or kill the proliferating cancer cells in culture, however this may not represent most therapy-resistant/adaptable cells. As a next step, therapies are often evaluated in xenograft models in mice. Although xenograft models appear to be better than *in vitro* cell culture with regard to modeling the cells that may matter more in the body, they too are not ideal for evaluating combination therapies that would be effective against a heterogeneous disease that does not respond to currently offered therapies. As an example, if one creates a xenograft model of a highly heterogeneous TNBC using a cell line (or with a patient-derived tumor) and treats with a chosen single agent, it would be the norm to encounter resistance to therapy. However, these models do not offer the flexibility of evaluating effective combination therapies that would eradicate most therapy-resistant (panresistant) relatively rare cancer cells that drive therapy resistance and cancer evolution. A big part of the problem is that there is only a limited time for evaluating anti-metastasis therapies in mice since they need to be sacrificed before primary tumor grows to a large/painful stage.

We have proposed an alternative strategy that relies on modeling panresistant cancer cells *in vitro*, which could be very useful for evaluating therapies [8]. To mitigate the limitations of cell culture, first we chose to model the panresistant TNBC cancer cells from the SUM149 cell line that has been established from an aggressive Inflammatory Breast Cancer (IBC). In this regard, IBC is comprised of all the clinically definable subgroups that are present in non-IBC; however, IBC is a lot more aggressive disease than non-IBC [10, 11]. The second element in our strategy is a harsh body-like selection for enriching resistant cells: less than 0.01% cells in population survive a long-term metabolic challenge; such metabolic challenges drive cancer evolution in the body. We have evidence that the metabolically adaptable (MA) cells that survive and grow in a glutamine-deficient culture medium efficiently metastasize to multiple organs from fat pad xenografts in nude mice, feature embryo-like gene expression, and are a good model of panresistant cancer cells [7, 8]. A comparative long-term evaluation of anticancer agents on MA cells versus parental SUM149 cell line yields information that could be useful in predicting impending resistance to therapy. We discovered that the *FTO* gene that encodes an RNA demethylase, which regulates fatness and obesity [12], is amplified in MA cells [8].

Obesity is a risk factor for the occurrence of a variety of cancers, including breast cancer, and it often predicts poor outcomes [13]. In general, obesity is a result of a metabolic state favoring energy storage over energy utilization. A variety of genomic alterations, e.g., in *FTO* or the genes that functionally interact with it, could confer susceptibility for obesity. FTO plays a key role in energy balance at both organismal and cellular levels [14–16]. Evolution has selected variants in the *FTO* gene that permit survival under metabolic scarcity [14]. Furthermore, genome wide association studies have identified *FTO* as being strongly associated with estrogen receptor-negative breast cancer, including TNBC [17]. We hypothesized that the FTO protein and its partners that promote obesity may help in the survival of SUM149-MA TNBC cells under metabolic scarcity (prolonged lack of glutamine in medium) that kills 99.99% of SUM149 cells. A similar selective advantage that obesity-related molecular networks could confer on cancer cells in the body may provide an explanation for linkage between obesity and cancer.

Recently it has been reported that a long-range interaction between intron 1 of *FTO* and enhancer of the homeobox gene *IRX3* governs *IRX3* gene expression, thus influencing cell fate decisions leading to obesity [18, 19]. Here we report that IRX3 is significantly overexpressed in SUM149-MA cells as compared to the parental SUM149 cell line, supporting our hypothesis. Further studies aimed at exploring the potential mechanisms that could help rare progenitor-like breast cancer cells survive metabolic challenges, indicated the involvement of several regulators of energy balance such as ARID5B, IRX5, and CUX1 P200 repressor besides FTO and IRX3. To determine the functional significance of FTO protein, we utilized a pharmacological inhibitor MO-I-500. These results further support the concept that FTO is important in the survival of rare embryo-like SUM149 TNBC cells when they face a severe metabolic challenge, e.g., a prolonged lack of glutamine.

## Results and Discussion

### Overexpression of IRX3 in SUM149-MA Cells

**Fig 1:**
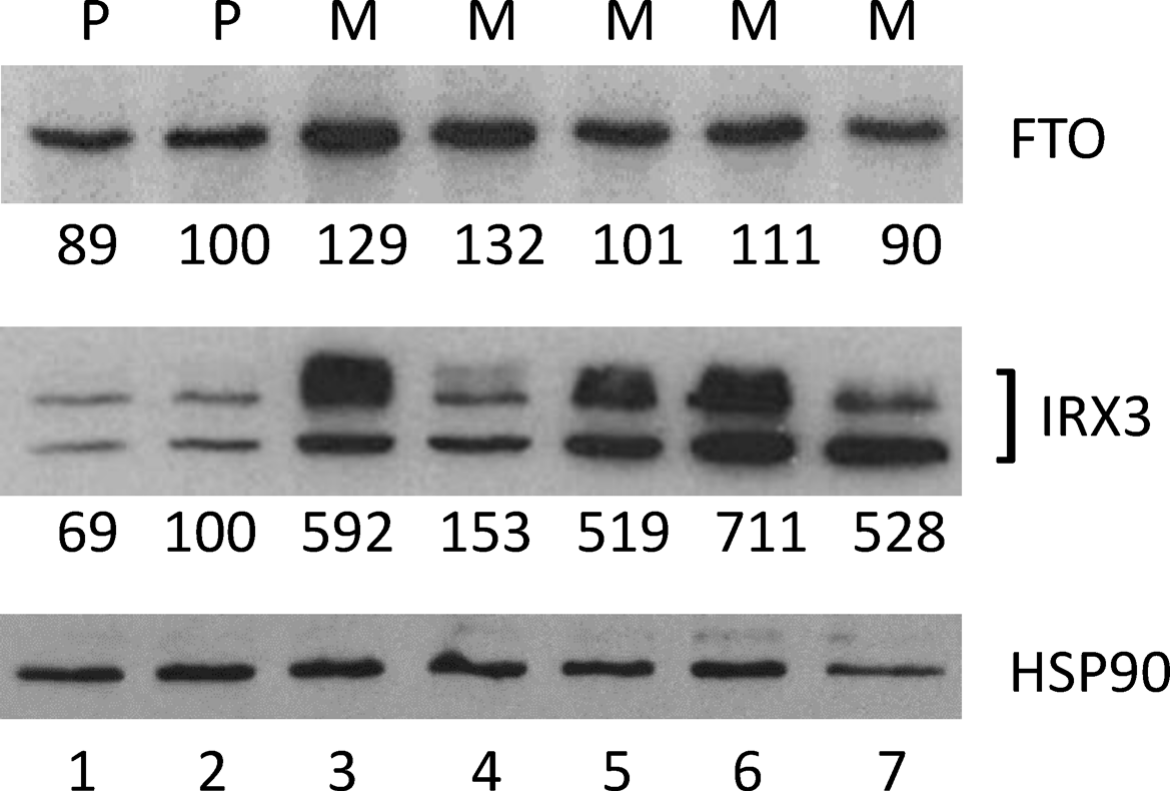
Overexpression of IRX3 Protein in MA Cells. We performed western blotting parallel to detect FTO and IRX3 proteins. The IRX3 protein appears as 2–3 bands. The cell lysates were from the parental SUM149-Luc cell line cultured in complete medium (lane 1), SUM149-Luc cell line cultured in a medium containing dialyzed fetal bovine serum for 3 passages (lane 2), MA cell line maintained in Gln-free medium for 4 passages (lane 3), MA1 cell line maintained in Gln-free medium for 12 passages (lane 4), MA cell line maintained in Gln-free medium for 9 passages followed by culture in glutamine-containing medium for 4 passages (lane 5), MA cell line maintained in Gln-free medium for 2 passages followed by culture in glutamine-containing medium for 7 passages (lane 6), or MA cell line maintained in Gln-free medium for 9 passages (lane 7). We re-probed the filters with an HSP90 antibody as a gel loading and protein transfer controls; re-probe of the IRX3 blot is shown. Relative intensities of bands are shown at the bottom of panels; lane 2 is designated as the parental cell line control (100% value) since it represents the cells growing in a similar medium as MA cell cultures (lanes 3–7). P, parental cell line; M, MA or MA1 cell line.

We have previously reported that the rare SUM149 cancer cells that survive a prolonged lack of glutamine in culture medium carry a *FTO* gene amplification on chromosome 16 q12.2; the amplified region includes *RBL2*, *AKTIP*, *RPGRIP1L*, and *FTO* genes [8]. We also observed a corresponding increase in FTO protein by western blotting [8]. According to recent reports, the *FTO* locus controls energy balance not only through the alpha-ketoglutarate-dependent RNA demethylase activity of FTO protein, but also through a long-range chromatin interaction between *FTO* and *IRX3* loci [18, 19]. This leads to IRX3 overexpression; abnormal IRX3 expression is an important driver of obesity and type 2 diabetes. Both *FTO* and *IRX3* genes are located on chromosome 16 at approximately 500 kilo base distance from each other. In two separate experiments involving selection of metabolically adaptable SUM149 cells, we detected similar gene amplification of the region around *FTO* with a comparative genomic hybridization (CGH) array [8]. Significantly, the *IRX3* gene is not amplified in SUM149-MA cells [8]. We noticed in our gene expression microarray data that SUM149-MA cells produce more IRX3 mRNA than the parental SUM149 cell line [8]. Considering the important role of IRX3 in obesity, we determined the level of IRX3 protein in SUM149-MA cells and found it to be significantly elevated as compared to the parental SUM149 cell line (Fig 1). The magnitude of increase in protein expression was significantly more for the IRX3 protein as compared to the FTO protein (compare top and middle panel in Fig 1). Fig 1 includes western blot data obtained with 3 different batches of MA cells (originating from 2 different selections in glutamine-free medium) cultured in glutamine-free medium plus 2 different batches of MA cells after the cells were switched back to glutamine containing medium. Overall these results strongly suggest that the IRX3 protein is overexpressed in MA cells in a reproducible manner, and that higher than basal level (the levels in parental SUM149 cells) of IRX3 protein persists even after the cells are switched back to glutamine-containing medium.

Our results are consistent with the emerging model in which activation of transcription in one locus, e.g., *FTO*, could open up a super-enhancer such as the one located in intron 1 of the *FTO* gene, which then activates transcription at other sites in chromatin. In this particular example, although the *IRX3* gene is at a 500 kb distance from the *FTO* gene, they would topographically co-localize for the activation of transcription of *IRX3*. Based on our CGH array data that *FTO* gene is amplified by a factor of 0.5, it is unlikely that both *FTO* alleles are amplified in all SUM149-MA cells [8]. We can expect both unamplified and amplified versions of *FTO* to be situated approximately 500 kb away from the *IRX3* gene. We do not yet know whether unamplified, amplified, or both versions of *FTO* are responsible for the increase in IRX3 protein level. Nevertheless, increased expression of *IRX3*, along with gene amplification and overexpression of *FTO*, strongly support our hypothesis regarding the role of obesity-related molecular networks in TNBC. Interestingly, besides its roles in obesity and type 2 diabetes, IRX3 plays an important role in patterning in the embryo [20]. Since the SUM149-MA cells have been selected to survive under metabolic challenge, and they are also enriched for embryo-like gene expression, IRX3 may represent a link between these phenotypes.

### Potential Mechanisms of Energy Balance for Survival of Progenitor-like Breast Cancer Cells

**Fig 2:**
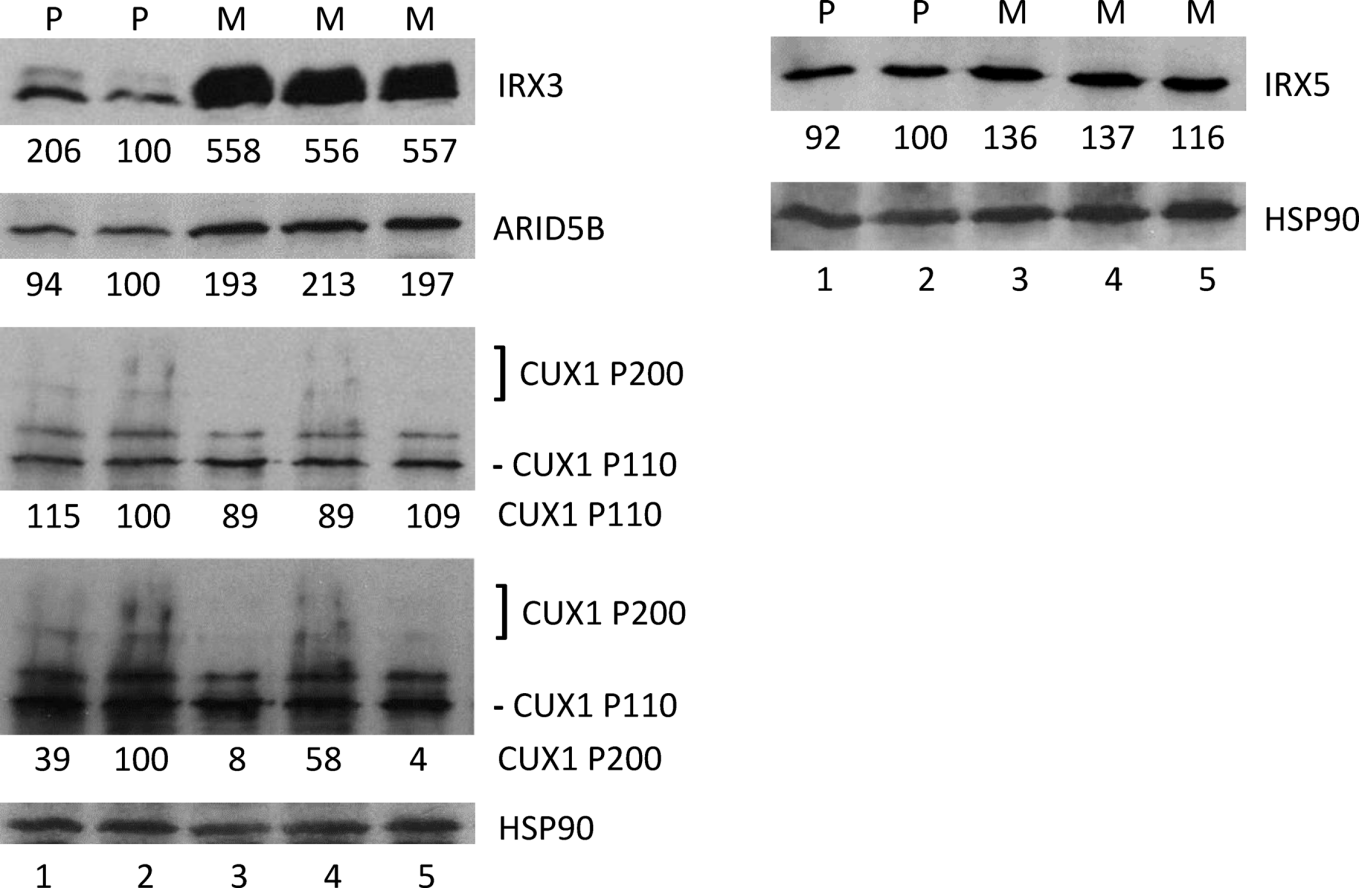
Detection of ARID5B, CUX1, and IRX5 Proteins in MA Cells. We performed western blotting to detect IRX3 (as a positive control), ARID5B, CUX1, and IRX5 proteins. We re-probed the filters with an HSP90 antibody as a gel loading and protein transfer controls. The HSP90 blot in the left panel is the re-probe of the ARID5B blot; the HSP90 blot the right panel is the re-probe of the IRX5 blot. The cell lysates were from the parental SUM149-Luc cell line cultured in complete medium (lane 1), SUM149-Luc cell line cultured in a medium containing dialyzed fetal bovine serum for 4 passages (lane 2), MA cell line maintained in Gln-free medium for 9 passages (batch 1; lane 3), MA cell line maintained in Gln-free medium for 2 passages followed by culture in glutamine-containing medium for 7 passages (lane 4), or MA cell line in Gln-free medium for 9 passages (batch 2; lane 5). Two exposures of the CUX1 blot are shown: a light exposure to optimally visualize the P110 variant of CUX1, and a dark exposure to optimally visualize the P200 variant of CUX1 (this one seen as a doublet). Relative intensities of bands are shown at the bottom of panels; lane 2 is designated as the parental cell line control (100% value) since it represents the cells growing in a similar medium as MA cultures (lanes 3–5). P, parental cell line; M, MA cell line.

An increased *FTO* gene dosage could independently provide survival advantage under metabolic challenge by shifting to a metabolic state that relies on less energy expenditure. In addition, cis-acting elements present in the first intron of *FTO* could influence the expression of other genes involved in energy balance, e.g., *IRX3*. We base our interpretation of *IRX3* overexpression in embryo-like MA cells on the studies showing a chromatin architecture-based long-range interaction between *IRX3* enhancer and *FTO* intron 1 [18, 19]. Investigating the mechanism through which rs1421085 SNP located on intron 1 of *FTO* influences obesity in persons of European ancestry, Claussnitzer et al. [19] reported that the obesity-associated C allele disrupts interaction of transcriptional repressor ARID5B leading to derepression of *IRX3* gene. Pertaining to the genotype of SUM149 cell line in this regard, we have recently performed next generation whole genome DNA sequencing at an average 62.5X coverage and found it to contain T non-obesity allele of rs1421085 (S1 Fig; we will report analysis of NGS data in a separate study). This cell line is derived from an African American woman; other SNPs (but not rs1421085) in intron 1 of *FTO* drive obesity in persons of this ethnicity [21]. To explore the potential mechanism of *IRX3* overexpression of MA cells, we analyzed relative level of ARID5B protein by western blotting. We found that MA cells possess a higher level of ARID5B than parental cell line (Fig 2); these data are consistent with gene expression microarray data (Table S2 in [8]). It is known that ARID5B can serve as a repressor or as an activator depending upon cellular context [22]. We favor the possibility that ARID5B serves as an activator of IRX3 transcription in MA cells.

Other activators and repressors beside ARID5B likely participate in obesity control. As an example, Cut-like homeobox 1 (CUX1) P200 isoform binds to its consensus binding site AATCAATA located on intron 1 of *FTO* to repress expression of both *FTO* and the adjacent gene *RPGRIP1L* in hypothalamus [23]. A single base change in this sequence as a part of obesity associated allele of SNP rs8050136 affects binding of CUX1 P200 repressor [23]. Apart from being associated with obesity, the rs8050136 SNP also appears to increase the risk of certain cancers [24]. We noticed that both obesity-associated A allele and normal C allele of rs8050136 are present in SUM149 cell line (S2 Fig). Interestingly, we found that the protein level of CUX1 P200 repressor is significantly reduced in MA cells growing in glutamine-free medium as compared to parental SUM149 cell line (Fig 2), which could be a potential mechanism of FTO overexpression from the obesity allele. We also found that the protein level of the P110 isoform of CUX1, which serves as an activator of FTO expression through preferential binding to non-obesity allele of rs8050136 [23], remained unchanged in MA cells as compared to the parental SUM149 cell line (Fig 2). This pattern of expression of CUX1 isoforms would potentially increase FTO expression of the obesity-associated allele but not that of the normal allele in MA cells.

Results from the CUX1 blot also indicate that the P200 repressor, which is not expressed (or expressed at a very low level) in MA cells maintained in glutamine-free medium, is expressed when the MA cells are maintained in glutamine-containing medium (compare lanes 3 and 5 with lane 4 in Fig 2). This result exemplifies how embryo-like cancer cells can adjust their metabolic state depending upon the availability of nutrients.

We have observed that once selected to survive lack of glutamine, the MA cells maintain their metabolic phenotype even when cultured in glutamine-containing medium. Majority of cells are able to survive a subsequent metabolic challenge in the form of a lack of glutamine ([7, 8], our unpublished data). This is not to say that expression of proteins does not change under conditions of glutamine availability. We find that protein levels of variety of proteins are influenced, some proteins affected more than others, upon availability of glutamine. We believe that MA cells growing in glutamine-free medium can generate sufficient glutamine for essential cell functions (e.g., availability for protein synthesis) by re-adjusting metabolism through alterations in enzyme levels [7] and possibly allosteric regulation of relevant metabolic enzymes, they still lack sufficient glutamine for non-essential but important functions thus maintaining a starvation-like metabolic state. One feature of starvation-like state would be a higher *FTO* expression; CUX1 P200 upregulation accompanied with *FTO* repression would be expected to occur when nutrients including glutamine are plentiful. Our results in Fig 2 are consistent with this working model.

We noticed that the frequency of C allele of rs8050136 is significantly higher in MA as compared to SUM149-Luc cell line (S2 Fig). Based on a total 104 reads from SUM149-Luc DNA and 94 reads from MA DNA, the C allele is present at 49% frequency in SUM149-Luc and at 62% frequency in MA. This result leads us to suggest that the C allele of *FTO* may be preferentially amplified in MA cells. It appears reasonable to speculate that there may be two alternative mechanisms for increasing FTO function- 1) increased gene dosage of the C allele, and 2) increased expression of the A allele via a reduction in the level of CUX1 P200 repressor under a severe and prolonged glutamine deficiency (as explained above). These two mechanisms need not be mutually exclusive; further studies will be required for providing a direct proof for these mechanisms.

It may be noteworthy that the non-risk allele of rs1421085 mentioned above (which is present in SUM149 cells) is part of the consensus binding site for CUX1 repressor; this potential interaction would be lost in case of the risk allele [25]. Therefore, a lack of P200 CUX1 in MA cells could also potentially contribute to derepression of the genes whose expression relies on chromatin regulation at this locus. At this time one can only speculate whether the genes in question could include *IRX3/IRX5* as well.

Finally, since obesity-associated SNP rs1421085 derepresses both IRX3 and IRX5 genes in pre-adipocytes, we wanted to know whether IRX5 is also overexpressed in MA cells. We found by western blotting that IRX5 is expressed in SUM149 cells and its level is 30–40% higher in MA cells than the parental cell line (Fig 2). This result indicates that expression of IRX3 and IRX5 may be co-regulated in MA cells similar to pre-adipocytes. We noticed that the increase in IRX5 protein level in MA cells is modest as compared to 5–6 fold increase in IRX3 protein level (Fig 2). We speculate that besides a common mode of co-regulation of these two genes at transcription level, there may be additional regulatory mechanisms that may be responsible for the lack of similarity in fold increase in IRX3 versus IRX5 proteins in MA cells.

Since the 90 kb-long obesity-associated region in *FTO* intron 1 has many cis-acting elements that could interact with a variety of transcriptional regulators (which may differ depending upon the cell type), mechanisms of energy balance could differ between adipocytes and cancer cells in our model. Further studies are required to investigate the mechanisms of energy balance in most adaptable rare TNBC cells within a highly heterogeneous disease. Nevertheless, our results reveal possibly meaningful relationships between metabolic adaptability, *FTO/IRX3* expression, and embryonic phenotype in our *in vitro* model of therapy-resistant TNBC. The novelty of our approach lies in being able to enrich therapy resistant rare embryo-like cancer cells based on their adaptable metabolic state rather than commonly used approaches of generating abnormal cancer cells through manipulation of specific gene products.

### Role of FTO in SUM149 Cells Facing a Metabolic Challenge

To determine whether the RNA demethylase activity of FTO protein has an important function in SUM149 cells, we utilized a pharmacological inhibitor of FTO called MO-I-500 [26]. We favor this strategy for a speedy translation in clinic. The use of a small molecular weight inhibitor also allows us to enforce inhibition of FTO in all subpopulations of cells in a heterogeneous cell line for prolonged periods as compared to the commonly used siRNA approach. We designed MO-I-500 as an alpha-ketoglutarate mimic, which has shown remarkable specificity towards FTO; MO-I-500 was referred as compound 7d in that study [26]. It inhibits FTO *in vitro*, and importantly *in* vivo. The IC50 for MO-I-500 is 8.7 μM for the inhibition of purified FTO demethylase catalyzing demethylation of an artificial small methylated substrate [26]. MO-I-500 treated cells exhibited a global increase in RNA methylation; HeLa cells treated with 25 μM MO-I-500 for 24 hours showed a 9.3% increase in N^6^-methyl-adenosine content in total RNA [26].

**Fig 3:**
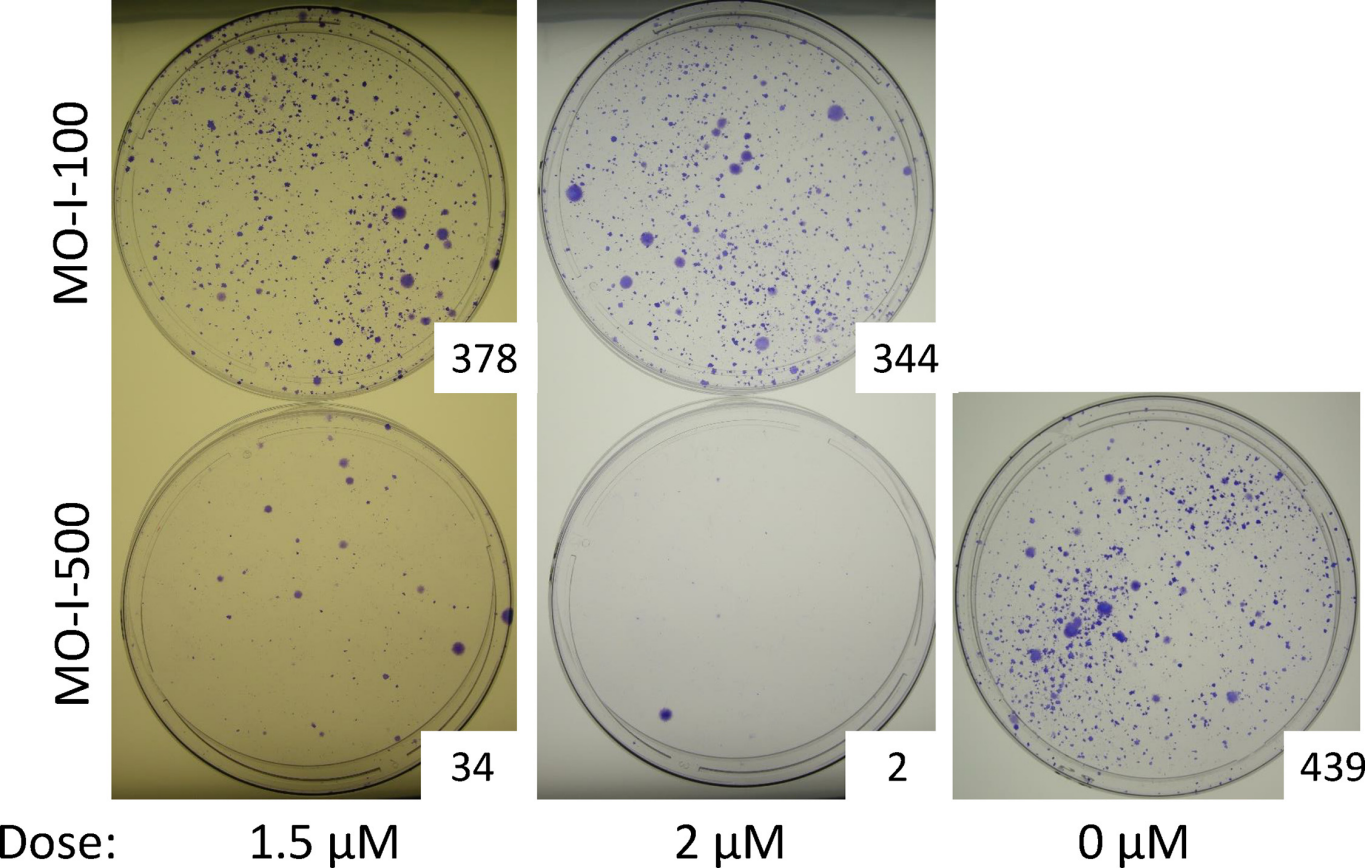
MO-I-500 Treatment During a Metabolic Challenge Diminishes Cell Survival. We plated SUM149-Luc cells with or without indicated doses of MO-I-500 (bottom panel), MO-I-100 (top panel), or DMSO solvent alone (0 dose), in a glutamine-free medium. We stained the colonies after 22 days of treatment in culture and photographed. The number of colonies, which were scored with ImageJ software, are shown at lower right of dishes.

First, to determine whether inhibiting FTO would affect the ability of the adaptable rare SUM149 subpopulation to survive and form colonies in a glutamine-deficient medium, we treated cells with MO-I-500 in this medium for 22 days and then stained the colonies. We observed a significant decrease in the number of colonies in MO-I-500-treated culture as compared to DMSO solvent-treated culture or the culture treated with a control compound MO-I-100 (Fig 3). MO-I-100 is structurally related to MO-I-500, but it does not inhibit FTO (see [26] for structures). In the experiment shown here, we used MO-I-500 up to 2 μM concentration, which severely inhibited survival and colony-forming ability of SUM149-Luc cells (compare plates shown in Fig 3). These results are consistent with FTO being important in cell survival under a metabolic challenge. We performed this experiment several times using different concentrations of compounds, different length of treatment, and different batches of cells. The result is reproducible in showing a dramatic decrease in the number of colonies upon MO-I-500 treatment. S3 Fig includes data from 3 such experiments. We have also included data from an experiment wherein 4 dishes each were set up together for treatment with 2.0 μM MO-I-500 and DMSO control. MO-I-500 treatment caused a dramatic (>95%) inhibition in colony formation as compared to the control group, which was statistically significant (p < 0.0001); the two-tailed p value was calculated with the unpaired *t*-test (S4 Fig).

Pertaining to the mechanistic insight as to why inhibition of FTO in glutamine-free medium kills or stops these cells from growing, more than 99% SUM149 cells die due to prolonged lack of glutamine in culture medium. So the phenomenon that we are describing may not apply to all cells but to those that survive a severe prolonged metabolic challenge. Our previous work has shown that the rare cells that survive not only possess metabolic adaptability (ability to survive a severe lack of nutrients) but also overall adaptability [7, 8]. Based on the gene expression data, an important feature of the MA cells is their embryo-like phenotype; they feature a low expression of GRHL2 and a high expression of ZEB1, which correlate with epithelial to mesenchymal transition and provide a mechanism for generating cancer stem cells [8]. Of importance from the perspective of current study, analysis of gene expression data revealed that molecular networks influencing lipid metabolism (and carbohydrate metabolism) are significantly altered in MA cells (see Table S3 in [8]).

Based on numerous studies on the role of FTO and IRX3 in obesity, it is evident that these master regulators of obesity regulate both metabolic state (energy expenditure versus energy conservation) and developmental reprogramming (e.g., converting mesenchymal adipocyte precursor to white adipocyte). Our data are consistent with the notion that in the rare embryo-like cancer cells FTO may be important in shifting energy balance in favor of preservation over expenditure (similar to its role in pre-adipocytes), and that this shift may be critical for their initial survival and proliferation without glutamine in the culture medium, leading to establishment of MA cell line ([8], this study). Unlike the rare cells that give rise to MA cell culture, most SUM149 cells in culture lack this plasticity in their metabolic state and regulatory state; they are overly dependent on glutamine and other nutrients. Various studies on nutrient sensing would suggest that prolonged lack of glutamine would trigger signaling pathways leading to cell death in majority of cancer cells. A simple way to explain our results obtained with MO-I-500 is that FTO inhibition affects the metabolic plasticity of the rare embryo-like cancer cells, which results in their inability to survive a severe and prolonged lack of glutamine. At this time we do not know which specific targets of FTO RNA demethylase and/or IRX3 transcription factor provide survival advantage when initially facing a metabolic challenge such as a lack of glutamine.

**Fig 4:**
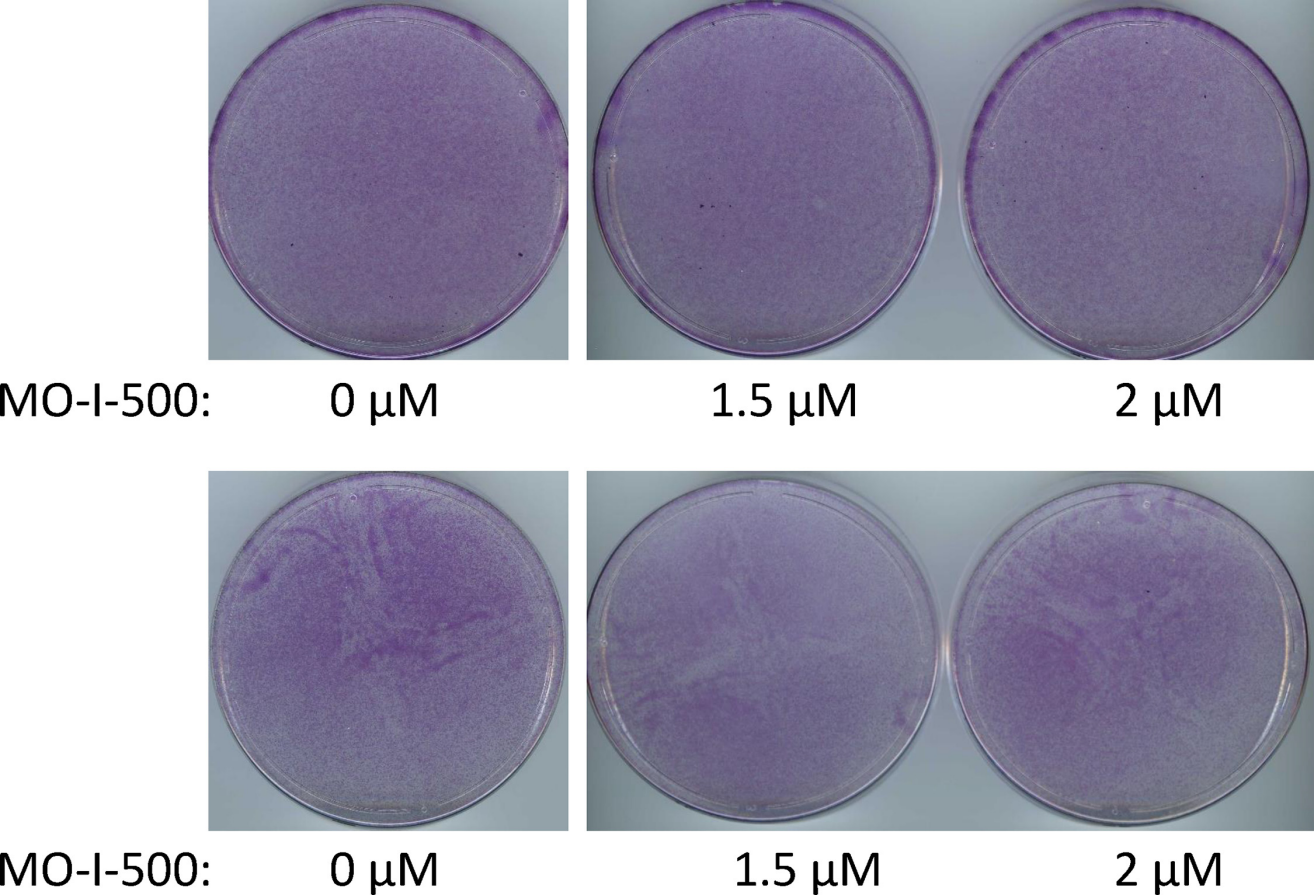
Relatively Little Effect of MO-I-500 on SUM149 Cells in Medium Containing Glutamine. We treated dishes with SUM149-Luc cells with indicated doses of MO-I-500 for 7 days in two different glutamine-containing media: complete medium (top) or glutamine-containing medium with dialyzed fetal bovine serum (bottom). Then we stained the dishes with crystal violet and scanned.

Next, to determine whether MO-I-500 would affect SUM149 cells in a regular culture medium that does not impose a metabolic scarcity, we treated the cells with the same concentrations of the inhibitor as above. We cultured cells in a regular culture medium containing glutamine or in a medium that contains dialyzed fetal bovine serum instead (this is another control since we use dialyzed fetal bovine serum in a glutamine-deficient medium in order to drastically lower glutamine level). We found that under either of these culture conditions, up to 2 μM MO-I-500 had essentially no effect on cell growth. The dishes become confluent in seven days, similar to DMSO solvent-treated dishes (compare plates shown in Fig 4). To determine whether MO-I-500 had any effect on cell growth in actively proliferating cells, we performed a similar experiment in a 96-well format and determined relative cell proliferation with MTS assay using CellTiter 96® AQueous One Solution Cell Proliferation Assay kit (Promega Corporation, Madison, WI). Based upon the MTS assays, treatment with 2 μM MO-I-500 (which is the highest concentration used in these experiments) caused a modest 20–25% inhibition in cell proliferation of the parental SUM149-Luc cell line in glutamine-containing medium (S5 Fig). These results are consistent with the cell staining data (Fig 4). These results imply that *in vitro* cell culture conditions, with plenty of carbon and nitrogen sources being present, may render FTO function less relevant. In other words, a low basal level activity of FTO may be sufficient for cell proliferation in these culture conditions. In this regard, it is important to note that *FTO* is not an absolutely essential gene since mice with homozygous deletion of *FTO* can be generated [27].

Our results indicate that FTO function is important for the survival of rare highly adaptable SUM149 cells in glutamine-free medium. It is noteworthy that the SUM149 cells selected in glutamine-free medium are capable of facing additional metabolic challenges as well, e.g., a total lack of glucose for weeks [7, 8]. Our results obtained with MO-I-500 are consistent with FTO being important under metabolic scarcity- 1) FTO expression goes up in a variety of cell types when a chicken is starved for a period of time [28], and 2) *FTO* variants promoting obesity provide survival advantage when sufficient food/nutrients are not available [14]. Analogous to the data from a starving chicken, wherein FTO level is low in tissues of an un-starved animal, we have also noticed that FTO protein level goes down when SUM149-MA cells are cultured in a glutamine-containing complete medium [8].

### Relatively Little Effect of FTO Inhibition in SUM149-MA Cells

**Fig 5:**
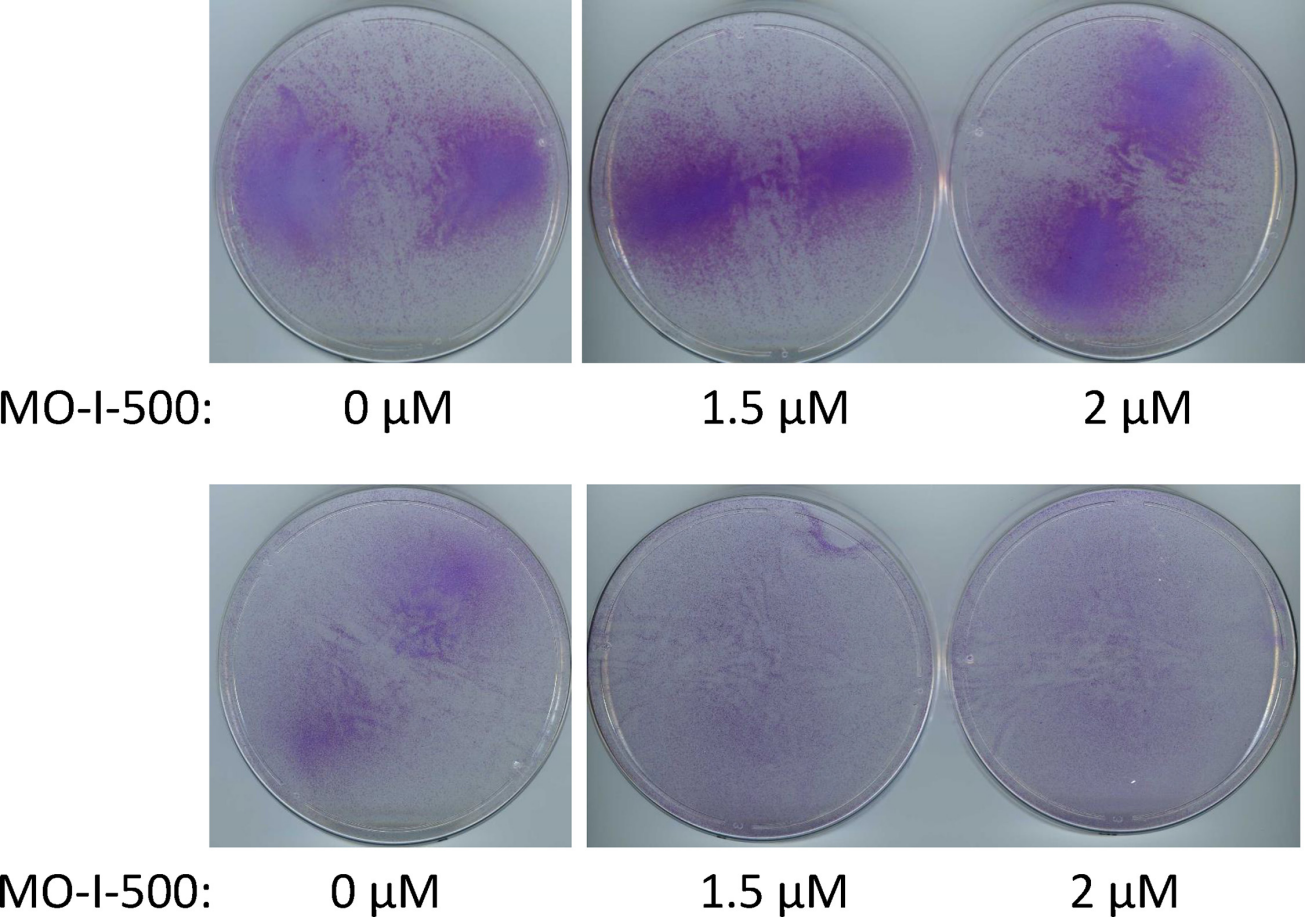
Relatively Little Effect of MO-I-500 on MA Cells. We treated dishes with SUM149-MA cells with indicated doses of MO-I-500 for 7 days in a glutamine-containing medium with dialyzed fetal bovine serum (top panel) or in a glutamine-free medium with dialyzed fetal bovine serum (bottom panel). Then we stained the dishes with crystal violet and scanned.

We asked whether MO-I-500 would affect highly adaptable SUM149-MA cells that have already been selected in glutamine-free medium and cultured for several passages, representing multiple cell divisions in this medium. We have previously shown that SUM149-MA cells are resistant to a variety of anticancer agents, and they could serve a good usable model of panresistance in cancer [8]. We observed that treatment of SUM149-MA cells with MO-I-500 failed to significantly inhibit their growth in complete medium (not shown) or in a medium containing glutamine and dialyzed fetal bovine serum (compare plates shown in Fig 5 top panel). Considering the similar results obtained with the parental SUM149 cell line (Fig 4), this result is not surprising.

Finally, we asked whether MO-I-500 would affect the SUM149-MA cell culture that has been continuously maintained for several passages in a glutamine-free medium. At this point the rare cells that succeed in facing the metabolic challenge have already been selected and expanded in culture. In the initial phase of culture in glutamine-free medium, we observe a heterogeneous response as surviving cells try to grow. Some cells may survive, but fail to yield progeny that would proliferate well. By the time we treated SUM149-MA cells with MO-I-500, or used them for a variety of other experiments, the cells were growing well, albeit a bit slower as compared to the parental cell line in complete medium. MO-I-500 treatment of SUM149-MA cells that were continuously cultured in a glutamine free medium did not significantly affect their growth as cells in all dishes grew to confluency in 7 days (compare dishes in Fig 5 bottom panel). It appears that the MO-I-500 treatment which is effective at the time of initial selection of MA cells is no longer effective after the MA cells have been selected and cultured in a glutamine-deficient medium (compare Fig 3 and Fig 5 bottom panel). As reported previously, MA cells are resistant to a variety of known and experimental anticancer agents [8]. Emergence of MA cells under a selection pressure reflects the nature of therapy resistance in TNBC, which is even more pronounced in triple-negative Inflammatory Breast cancer.

To determine whether MO-I-500 had any effect on cell growth in actively proliferating MA cells, we performed a similar experiment in a 96-well format and determined relative cell proliferation with MTS assay. The treatment did not affect cell proliferation of MA cells growing in glutamine-free medium, and 2 μM MO-I-500 affected their proliferation by a modest 11% in glutamine-containing medium (S6 Fig). These results are consistent with the cell staining data presented in Fig 5.

Our data indicate that FTO function is important during the initial selection of embryo-like MA cells under a metabolic challenge, i.e., a lack of glutamine. They do not rule out the role of FTO in cells growing in regular culture medium. One important goal of this study is to explore whether MO-I-500 could be useful in overcoming therapeutic resistance in TNBC; therefore, we chose relatively low concentrations of MO-I-500 for the experiments so that the results of our *in vitro* model would have a higher likelihood of predicting response in patients. Typically, high concentrations of drugs taken over time increase the likelihood of side effects, and high drug concentrations are not achievable for several reasons in metastatic breast cancer. Our experiments were designed for comparative evaluation of low-dose MO-I-500 with parental SUM149 and MA cell lines. Having said that, we have data showing that high 10 to 20 μM concentrations of MO-I-500 kill essentially all cells in both parental SUM149 and MA cell lines even in glutamine containing medium after several days of treatment (data not shown). These results may indicate that FTO serves an essential cellular function in SUM149 cell line under the regular cell culture conditions.

**Fig 6:**
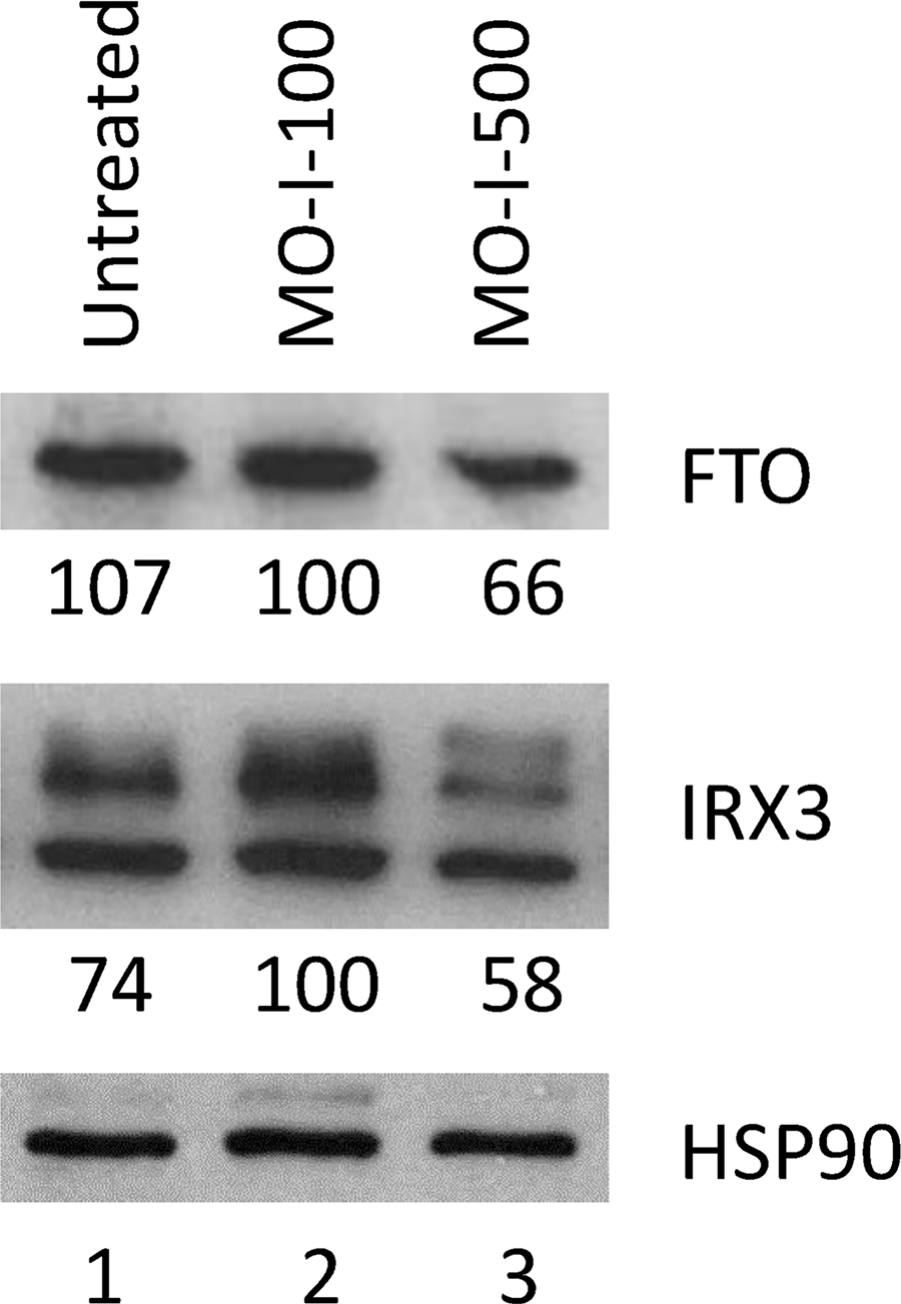
Reduced FTO and IRX3 Protein Levels Upon MO-I-500 Treatment of SUM149 Cells in Glutamine-Free Medium. The cell lysates from the SUM149-Luc cells treated with 1.25 μM MO-I-100 (lane 2) or 1.25 μM MO-I-500 (lane 3) in Gln-free medium for 35 days were subjected to western blotting as described in Materials and Methods. Lane 1 was loaded with the lysate from control untreated SUM149-Luc cells after culturing them in glutamine-free medium for 35 days. Western blotting was performed in parallel to detect FTO and IRX3 proteins. We re-probed the filters with an HSP90 antibody as a gel loading and protein transfer controls; re-probe of the IRX3 blot is shown. Relative intensities of bands are shown at the bottom of panels; lane 2 is designated as the control (100% value) since it represents the cells growing under similar conditions as the experimental cells (lane 3).

We were curious to see whether MO-I-500 treatment affects the level of FTO and IRX3 proteins in the subpopulation SUM149 cells that initially survives in glutamine-free medium. For the feasibility of obtaining a sufficient number of cells in this medium for western blot analysis after a long treatment with MO-I-500, we used it at 1.25 μM, which is suboptimal for the inhibition of colony formation (e.g., see Fig 3). Western blot analysis showed that the MO-I-500 treatment reduced the cellular levels of both FTO and IRX3 proteins by approximately 34% and 42%, respectively, under these conditions as compared to the MO-I-100-treated cells in parallel (compare lanes in Fig 6). As a possible explanation of these results, MO-I-500 binding could enhance FTO protein degradation, which would lead to decreased demethylation of obesity-related RNAs including IRX3 mRNA, thus reducing synthesis of IRX3 protein. Alternatively, as MO-I-500 treatment would possibly eliminate/disadvantage the subpopulation of cancer cells that rely on high levels of FTO and IRX3 proteins for their initial survival and growth in glutamine-free medium, the cells with lower levels of FTO and IRX3 would emerge during the 5 weeks treatment of a very heterogeneous cell population. Further studies are required for understanding the molecular mechanisms for these results, and for determining whether these results are a part of MO-I-500 response or a part of resistance to MO-I-500.

### Importance of Energy Balance in the Evolution of Triple-Negative Breast Cancer

**Fig 7:**
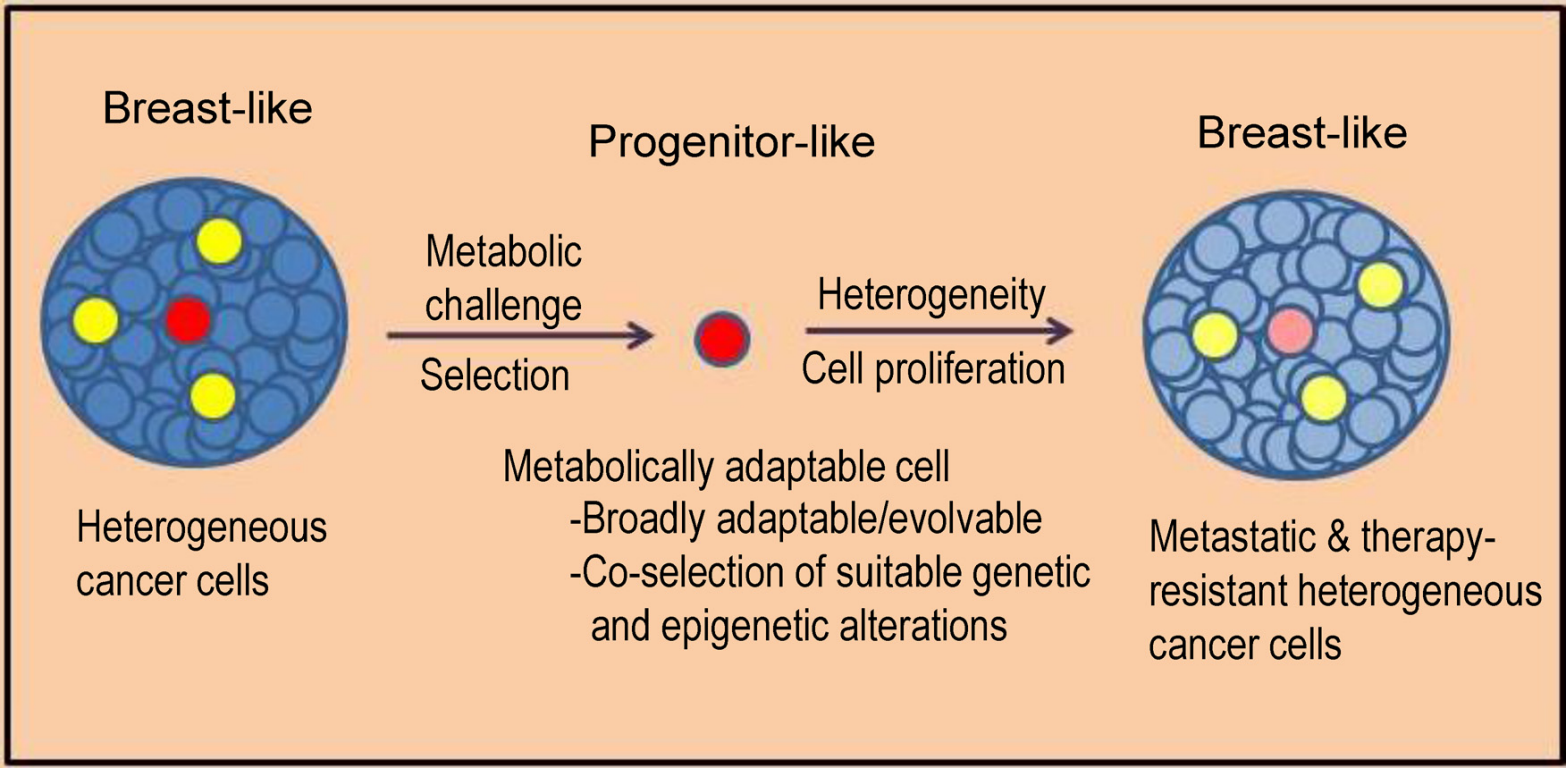
A Model Depicting a Snapshot of Evolution-like Process of Breast Cancer. Cancer progresses through an evolution-like process, which involves selection of adaptable cells under various challenges in the body including therapeutic interventions. A two-way linkage between metabolic state and regulatory state may allow enrichment of fittest cancer cells under metabolic challenges. Our approach involving a challenge of prolonged lack of glutamine in cell culture of a TNBC cell line is intended to enrich the fittest cells that would have a high likelihood of “evolving” to yield metastasis. Our results support this model and suggest that a severe metabolic challenge applied in a setting of high cellular heterogeneity would select rare progenitor-like cells from the majority breast-like cells; it would also co-select genetic and epigenetic alterations that would provide survival advantage in facing a variety of challenges in an evolution-like process ([8], this study). Our results support that progenitor-like cancer cells may utilize energy conservation as a strategy for survival under severe metabolic challenge. We adapted this model from the model presented as Figure 6 in [8].

Although we use knowledge gained from well-defined systems regarding the mechanisms of energy homeostasis, e.g., the one involving chromatin architecture-based interactions between *FTO* and *IRX3/IRX5* to interpret our data, we recognize that breast cancer cells differ from adipocytes. To elaborate on the hypothesis, a prolonged lack of glutamine, which kills more than 99% cells in our system, represents a major metabolic challenge. Cumulative evidence suggests that surviving cells not only have highly adaptable metabolic state but they are generally adaptable/evolvable and resistant to chemotherapeutic drugs (see model in Fig 7). Cancer cells use several strategies to survive shortage of critical nutrients, including autophagy, and non-traditional metabolism of extracellular and intracellular materials to meet their needs. We propose that an important component of survival strategy under a severe long-term challenge, e.g., a lack of glutamine, would be energy preservation through slowing of overall metabolism. Most cancer cells are not equipped to do so and therefore die; only rare embryo like cells have this metabolic plasticity. FTO and IRX3/IRX5, which regulate energy homeostasis, may help MA cells in this strategy for cell survival. We believe that the metabolic plasticity (being able to adapt according to nutrients supply) rather than a particular state of metabolism would be important in cancer evolution and therapeutic resistance.

It is important to consider that the features of the metabolic state that are important in cell survival under a severe metabolic challenge may not be of great value in cell proliferation in artificially rich culture medium [9]. For practical reasons most of our studies, particularly those involving analyses of gene expression and western blotting, are performed with cells that have been passaged in culture. The cell culture in complete medium may favor the most proliferative clones over the most adaptable clones. While recognizing these limitations, our model system can teach us a lot about the nature of roots of therapy-resistant TNBC, and help develop therapeutics against tumor adaptability.

### An *in vitro* Model of Adaptable TNBC Cells

Analogous to the cancer evolution process in the body, a metabolic challenge applied *in vitro* could select cells with an adaptable metabolic state, which could be a part of overall adaptable cellular state [8]. Our data suggest that SUM149-MA cells are endowed with high adaptability, which is derived from a variety of mechanisms. Our gene expression data points to not only metabolic alterations in MA cells that would permit their survival, but also the mechanisms that would generate genetic and epigenetic diversity- 1) cancer stem cell/embryonic phenotype, 2) cell cycle checkpoint/DNA repair defects, 3) genomic editing, and 4) chromatin modifications, to name a few [8]. Studies to investigate these specific mechanisms will continue in the future.

From the “evolution” perspective, the biological systems such as a cell line may have a natural tendency to maintain a large population of proliferative cells and a small population of adaptable cells if something were to go wrong. Our studies provide an insight into how therapy resistance could evolve under a severe metabolic scarcity, and suggest an approach to model the panresistant cells in a usable model for testing therapies. Specifically with regards to the FTO and associated molecular networks, our results would argue that the agents that affect obesity could be useful in slowing cancer evolution, particularly if they are introduced early. Perhaps more importantly, our results reveal the type of cells that are capable of driving cancer evolution and therapy resistance in a heterogeneous disease like TNBC. Our approach could be developed into a useful platform for testing combination therapies.

### SUM149-MA Cells as a Suitable Model for Testing Combination Therapies

Conceptually normal cells would be more adaptable than abnormal cells such as cancer cells. Therefore, modeling highly adaptable and highly abnormal human TNBC cells *in vitro* which could efficiently drive cancer evolution and therapeutic resistance similar to the one observed at metastasis stage is not trivial. Different types of selective pressures would eliminate most cancer cells, until highly abnormal and highly adaptable cancer cells overwhelm the body’s defenses. Our studies suggest that SUM149-MA cells are a good model of highly abnormal and highly adaptable cancer cells [8]. As a cautionary measure, it appears unlikely that the glutamine deficiency in a culture medium would select highly abnormal and highly adaptable cancer cells in all cancer cell lines or even in all TNBC cell lines. For example, a commonly used MDA-MB-131 TNBC cell line does not appear to have an adaptable metabolic state that would support long-term growth in glutamine-deficient medium [7].

We believe that the success of SUM149-MA cells as a model of panresistance relies on SUM149 cell line maintaining a small number of highly adaptable cells even in artificial cell culture conditions, and being able to select and investigate these cells. SUM149 cell line contains several genetic defects that are often seen in therapy-resistant TNBC, e.g., *BRCA1* mutation, gain of function mutation in *TP53*, and microdeletions in *PTEN*, to name a few [29, 30]; some of these defects are considered undruggable. Above all, SUM149 cells are capable of generating a tremendous cellular heterogeneity, and SUM149-MA appears to be even better in this regard. Therefore, SUM149-MA cell line would be a good *in vitro* model for evaluating the combination therapies needed for overcoming therapeutic resistance.

### Conclusions

There is a pressing need for simple and reliable models for testing potential therapeutic drugs that accurately predict how drugs will act in cancer patients. Our cell-based model of panresistance that involves function-based selection of rare but highly adaptable cells that drive cancer, as well as testing therapies in long-term assays, will make the test results more predictive of response and have a major impact on drug development and cancer care. Our approach addresses a real need in the clinic, where we must anticipate cancer evolution as therapies are applied and respond therapeutically in a timely manner. Despite the abundance of models, we lack suitable models for evaluating combination therapies that would be needed for overcoming therapeutic resistance in an evolving disease. Our applied approach to modeling the roots of therapy-resistance will complement other approaches to discovering safe and effective therapies for preventing cancer recurrence, and for treating metastasis. The data presented in this paper provides further support for the validity of our approach for modeling therapy resistance in TNBC.

## Materials and Methods

### Cell Lines and Culture

The SUM149 IBC cell line, originally obtained from Stephen Ethier (Barbara Ann Karmanos Cancer Institute, Detroit, MI, USA), was grown in Ham’s F-12 medium supplemented with 5% fetal bovine serum (FBS), 5 μg/ml of insulin, 1 μg/ml of hydrocortisone, 100 U/ml of penicillin, and 100 μg/ml of streptomycin in a humidified 5% CO_2_ atmosphere. We previously described SUM149-Luc, a luciferase-transfected cell line [31].

### Selection and Culture of MA Variants

We selected MA variants by plating 1 million SUM149-Luc cells in a glutamine-deficient medium containing dialyzed FBS. We recently described selection of rare variants (approximately 0.01% cells in population) and characterization of a cell culture established from these colonies [7, 8]. In the current study, we refer to this cell line as SUM149-MA or MA. All experiments in this study have been performed with the MA cell line which was referred as MA2 (established after selection from one million cells) in a previous study [8]. The MA1 cell line (established after selection from half million cells) in previous studies [7, 8] was included only for the data in Fig 1 in this study. MA cells can be passaged indefinitely in glutamine-deficient medium. However, to minimize the loss of cellular characteristics in cell culture, we investigated MA cells that were in a glutamine-free medium for less than 10 passages. Similarly, for investigating the MA cells in a glutamine-containing medium, we also cultured them for less than 10 passages in such medium.

### Western Blotting

We separated proteins by sodium dodecyl sulfate-polyacrylamide gel electrophoresis and detected various proteins by Western blotting as described previously [32]. The following primary antibodies were used for detection: anti-FTO (catalog number MABE227, EMD Millipore, Billerica, MA), anti-IRX3 (catalog number ab174307, Abcam, Cambridge, MA), anti-ARID5B (catalog number HPA015037, Sigma Chemical Company, St. Louis, MO), anti-IRX5 (catalog number ab56681, Abcam), and anti-CUX1 (catalog number ABE217, EMD Millipore. We used the ECL prime blocking agent (GE Healthcare Life Sciences, Piscataway, NJ) for blocking and Lumigen TMA-6 reagents for detection (Lumigen, Inc., Southfield, MI). We have previously reported that the level of beta-actin detected by western blotting, which is commonly used for normalizing protein loading, is reduced in MA cells as compared to the parental cell line. After testing several other commonly used proteins for this purpose, we chose HSP90 as a gel loading and protein transfer control; we used an HSP90 antibody (catalog number E289 from Cell Signaling Technology, Danvers, MA) for re-probing the blots. We performed each western blot at least twice; the representative blots are shown. We determined relative intensities of bands by the ImageJ software (National Institutes of Health, Bethesda, MD); we chose 15 pixel as the cut-off value in order to score small colonies as well.

### FTO Inhibitor MO-I-500

Synthesis and initial characterization of MO-I-500 has been previously described [26]. MO-I-500 was referred as Compound 7d {N-(3,4-Dihydroxy-5-(4-chlorophenyl)-2-furanyl)ethanesulfonamide} in that study [26]. Synthesis of the control compound MO-I-100, referred as Compound 5 {5-Amino-4-hydroxy-2-(4-chlorophenyl)-furan-3-one}, has also been described [26]. We dissolved both the compounds in DMSO as 100 mM stocks, and stored at -20 degree C in small aliquots. The stock solution was diluted in DMSO just before adding to the culture medium. DMSO volume was equal to 0.04% of the volume of the culture medium in control dishes as well as those receiving the compounds.

### Evaluation of Test Compounds *in vitro*

We typically plated 0.5 million cells per 10-cm dish in duplicate in culture medium without glutamine and with dialyzed fetal bovine serum (glutamine-free medium), with glutamine and with dialyzed fetal bovine serum (glutamine-containing medium), and with glutamine and regular fetal bovine serum (complete medium). We added the test compounds at the same time as the trypsinized cells were plated on the dishes. We examined the cultures under microscope, and stained with crystal violet when appropriate. If a treatment had an effect, we waited for the surviving cells to yield colonies (approximately 3 weeks) before staining and photographing or scanning stained dishes. To determine whether a test compound had any effect on cell growth in actively proliferating cells, we performed a similar experiment in a 96-well format and determined relative cell proliferation with MTS assay using CellTiter 96® AQueous One Solution Cell Proliferation Assay kit (Promega Corporation, Madison, WI).

## Supporting Information

S1 Fig A Lack of Obesity-associated Risk Allele in rs1421085 SNV in SUM149 Cell Line, Related to Fig 2. The whole genome DNA sequencing was performed with Illumina Genome Analyzer (Beckman Coulter Genomics) at an average 62.5X coverage. DNA sequences in binary alignment and map (BAM) format were aligned with the reference hg19 human genome in Integrative genomics Viewer (IGV version 2.3, Broad Institute, Boston, MA); SUM149-Luc (top), MA (middle), and hg19 reference (bottom). The T base included in the rs1421085 SNV, which renders it non-risk allele, is shown in the middle. Both SUM149-Luc and MA had only non-risk T allele.
(JPEG)

S2 Fig Detection of Both Normal and Obesity-associated Risk Alleles in rs8050136 SNV in SUM149 and MA Cell Line, Related to Fig 2. The DNA sequences were read and aligned with the reference hg19 human genome as in S1 Fig; SUM149-Luc (top), MA (middle), and hg19 reference (bottom). The C/A base included in the rs8050136 SNV, which renders it non-risk versus risk allele, is shown in the middle. Both SUM149-Luc and MA had both non-risk (C) and risk (A) alleles. However, the ratio of C:A was significantly different between the two cell lines: 49:51 in SUM149-luc versus 62:38 in MA.
(JPEG)

S3 Fig Effect of MO-I-500 Treatment on Cell Survival During a Metabolic Challenge, Related to Fig 3. We plated SUM149-Luc cells with or without indicated doses of MO-I-500, MO-I-100, or DMSO solvent alone (0 dose), in a glutamine-free medium. We treated cells for different lengths of time, then washed off the drugs with phosphate-buffered saline, and allowed them to recover in glutamine-free medium without any drug before staining the colonies. Results from three separate experiments are shown: Panel A, treatment time 14 days and recovery time 8 days; panel B, treatment time 14 days and recovery time 20 days; panel C, treatment time 21 days and recovery time 1 day (this experiment is part of the experiment that is shown in Fig 2). The number of colonies is shown below the dishes (A and B) or on the lower right side of dishes (C).
(PDF)

S4 Fig Effect of MO-I-500 Treatment on Cell Survival in Glutamine-free Medium, Related to Fig 3. We plated SUM149-Luc cells in quadruplicate in 10 cm dishes with 2 μM MO-I-500 or DMSO solvent alone (0 dose) in a glutamine-free medium. We treated cells for 24 days and then stained the colonies with crystal violet. We obtained images in a scanner (Epson). Average number of colonies in treated and control groups along with standard deviation, as determined by the ImageJ software, is shown at the bottom.
(PDF)

S5 Fig Effect of MO-I-500 on the Proliferation of SUM149-Luc Cells in Presence of Glutamine, Related to Fig 4. We incubated SUM149-Luc cells in 96-well plate along with MO-I-500 at the indicated concentrations in quadruplicate for 7 days, and then performed MTS cell proliferation assay. Controls were cells treated with DMSO alone (0 dose of MO-I-500). Relative average absorbance along with error bars representing standard deviation are shown. Panel A: cells growing in complete medium; panel B: cells growing in medium containing glutamine and dialyzed fetal bovine serum. The 100% absorbance values for DMSO-treated cells were 0.55 (panel A) and 0.35 (panel B).
(PDF)

S6 Fig Effect of MO-I-500 on the Proliferation of MA Cells in Presence or Absence of Glutamine, Related to Fig 5. We incubated MA cells in 96-well plate along with MO-I-500 at the indicated concentrations in quadruplicate for 7 days, and then performed MTS cell proliferation assay. Controls were cells treated with DMSO alone (0 dose of MO-I-500). Relative average absorbance along with error bars representing standard deviation are shown. Panel A: cells growing in medium containing glutamine; panel B: cells growing in medium lacking glutamine. The 100% absorbance values for DMSO-treated cells are 0.39 (panel A) and 0.28 (panel B). We performed this experiment with the MA cells that were at passage 3 in glutamine-free medium after the initial selection.
(PDF)

## References

[1] Nowell PC (1976) The clonal evolution of tumor cell populations. Science 194: 23–28. 959840 10.1126/science.959840

[2] Fidler IJ, Kripke ML (1977) Metastasis results from preexisting variant cells within a malignant tumor. Science 197: 893–895. 887927 10.1126/science.887927

[3] Talmadge JE, Fidler IJ (2010) AACR centennial series: the biology of cancer metastasis: historical perspective. Cancer Res 70: 5649–5669. 10.1158/0008-5472.CAN-10-1040 20610625 PMC4037932

[4] Shah SP, Roth A, Goya R, Oloumi A, Ha G, Zhao Y, et al (2012) The clonal and mutational evolution spectrum of primary triple-negative breast cancers. Nature 486:395–399. 10.1038/nature10933 22495314 PMC3863681

[5] The Cancer Genome Atlas Network (2012) Comprehensive molecular portraits of human breast tumours. Nature 490:61–70. 10.1038/nature11412 23000897 PMC3465532

[6] Wang Y, Waters J, Leung ML, Unruh A, Roh W, Shi X, et al (2014) Clonal evolution in breast cancer revealed by single nucleus genome sequencing. Nature 512:155–160. 10.1038/nature13600 25079324 PMC4158312

[7] Singh B, Tai K, Madan S, Raythatha MR, Cady AM, Braunlin M, et al (2012) Selection of metastatic breast cancer cells based on adaptability of their metabolic state. PLoS ONE 7: e36510 10.1371/journal.pone.0036510 22570721 PMC3343010

[8] Singh B, Shamsnia A, Raythatha MR, Milligan RD, Cady AM, Madan S, et al (2014) Highly adaptable triple-negative breast cancer cells as a functional model for testing anticancer agents. PLoS One. 9:e109487 10.1371/journal.pone.0109487 25279830 PMC4184880

[9] McKnight SL (2010) On getting there from here. Science 330: 1338–1339. 10.1126/science.1199908 21127243

[10] Van Laere SJ, Ueno NT, Finetti P, Vermeulen P, Lucci A, Robertson FM, et al (2013) Uncovering the molecular secrets of inflammatory breast cancer biology: an integrated analysis of three distinct affymetrix gene expression datasets. Clin Cancer Res. 19:4685–4696. 10.1158/1078-0432.CCR-12-2549 23396049 PMC6156084

[11] Masuda H, Baggerly KA, Wang Y, Iwamoto T, Brewer T, Pusztai L, et al (2013) Comparison of molecular subtype distribution in triple-negative inflammatory and non-inflammatory breast cancers. Breast Cancer Res. 15:R112 10.1186/bcr3579 24274653 PMC3978878

[12] Gerken T, Girard CA, Tung YC, Webby CJ, Saudek V, Hewitson KS, et al (2007) The obesity-associated *FTO* gene encodes a 2-oxoglutarate-dependent nucleic acid demethylase. Science 318:1469–1472. 17991826 10.1126/science.1151710 PMC2668859

[13] Jain R, Strickler HD, Fine E, Sparano JA (2013). Clinical studies examining the impact of obesity on breast cancer risk and prognosis. J. Mammary Gland Biol. Neoplasia 18:257–266. 10.1007/s10911-013-9307-3 24221746

[14] Fredriksson R, Hägglund M, Olszewski PK, Stephansson O, Jacobsson JA, Olszewska AM, et al (2008) The obesity gene, *FTO*, is of ancient origin, up-regulated during food deprivation and expressed in neurons of feeding-related nuclei of the brain. Endocrinology. 149:2062–2071. 10.1210/en.2007-1457 18218688

[15] Berulava T, Ziehe M, Klein-Hitpass L, Mladenov E, Thomale J, Rüther U, et al (2013) FTO levels affect RNA modification and the transcriptome. Eur J Hum Genet. 21:317–323. 10.1038/ejhg.2012.168 22872099 PMC3573201

[16] Gulati P, Cheung MK, Antrobus R, Church CD, Harding HP, Tung YC, et al (2013) Role for the obesity-related *FTO* gene in the cellular sensing of amino acids. Proc Natl Acad Sci U S A. 110:2557–2562. 10.1073/pnas.1222796110 23359686 PMC3574930

[17] Garcia-Closas M, Couch FJ, Lindstrom S, Michailidou K, Schmidt MK, Brook MN, et al (2013) Genome-wide association studies identify four ER negative-specific breast cancer risk loci. Nat Genet. 45:392–398. 10.1038/ng.2561 23535733 PMC3771695

[18] Smemo S, Tena JJ, Kim KH, Gamazon ER, Sakabe NJ, Gómez-Marín C, et al (2014) Obesity-associated variants within FTO form long-range functional connections with IRX3. Nature. 507:371–375. 10.1038/nature13138 24646999 PMC4113484

[19] Claussnitzer M, Dankel SN, Kim KH, Quon G, Meuleman W, Haugen C, et al (2015) *FTO* Obesity Variant Circuitry and Adipocyte Browning in Humans. N Engl J Med. 373:895–907. 10.1056/NEJMoa1502214 26287746 PMC4959911

[20] Bellefroid EJ1, Kobbe A, Gruss P, Pieler T, Gurdon JB, Papalopulu N (1998) Xiro3 encodes a Xenopus homolog of the Drosophila Iroquois genes and functions in neural specification. EMBO J. 17:191–203. 9427753 10.1093/emboj/17.1.191 PMC1170370

[21] Jacobsson JA, Schiöth HB, Fredriksson R (2012) The impact of intronic single nucleotide polymorphisms and ethnic diversity for studies on the obesity gene FTO. Obes Rev. 13:1096–1109. 10.1111/j.1467-789X.2012.01025.x 22931202

[22] Yamakawa T, Sugimoto K, Whitson RH, Itakura K (2010) Modulator recognition factor-2 regulates triglyceride metabolism in adipocytes. Biochem Biophys Res Commun. 391:277–281. 10.1016/j.bbrc.2009.11.049 19913508

[23] Stratigopoulos G, LeDuc CA, Cremona ML, Chung WK, Leibel RL (2011) Cut-like homeobox 1 (CUX1) regulates expression of the fat mass and obesity-associated and retinitis pigmentosa GTPase regulator-interacting protein-1-like (RPGRIP1L) genes and coordinates leptin receptor signaling. J Biol Chem. 286:2155–2170. 10.1074/jbc.M110.188482 21037323 PMC3023512

[24] Hernández-Caballero ME, Sierra-Ramírez JA (2015) Single nucleotide polymorphisms of the FTO gene and cancer risk: an overview. Mol Biol Rep. 42:699–704. 10.1007/s11033-014-3817-y 25387436

[25] Peters U, North KE, Sethupathy P, Buyske S, Haessler J, Jiao S, et al (2013) A systematic mapping approach of 16q12.2/FTO and BMI in more than 20,000 African Americans narrows in on the underlying functional variation: results from the Population Architecture using Genomics and Epidemiology (PAGE) study. PLoS Genet. 9:e1003171 10.1371/journal.pgen.1003171 23341774 PMC3547789

[26] Zheng G, Cox T, Tribbey L, Wang GZ, Iacoban P, Booher ME, et al (2014) Synthesis of a FTO inhibitor with anticonvulsant activity. ACS Chem Neurosci. 5:658–565. 10.1021/cn500042t 24834807 PMC4140589

[27] Fischer J, Koch L, Emmerling C, Vierkotten J, Peters T, Brüning JC, et al (2009) Inactivation of the Fto gene protects from obesity. Nature 458:894–898. 10.1038/nature07848 19234441

[28] Tiwari A, Krzysik-Walker SM, Ramachandran R (2012) Cloning and characterization of chicken fat mass and obesity associated (Fto) gene: fasting affects Fto expression. Domest Anim Endocrinol. 42:1–10. 10.1016/j.domaniend.2011.08.001 22019092

[29] Chao HH, He X, Parker JS, Zhao W, Perou CM (2012) Micro-scale genomic DNA copy number aberrations as another means of mutagenesis in breast cancer. PLoS One. 7:e51719 10.1371/journal.pone.0051719 23284754 PMC3524128

[30] Barnabas N, Cohen D (2013) Phenotypic and molecular characterization of MCF10DCIS and SUM breast cancer cell lines. Int J Breast Cancer 2013:872743 10.1155/2013/872743 23401782 PMC3562669

[31] Singh B, Cook KR, Martin C, Huang EH, Mosalpuria K, Krishnamurthy S, et al (2010) Evaluation of a CXCR4 antagonist in a xenograft mouse model of inflammatory breast cancer. Clin Exp Metastasis 27: 233–240. 10.1007/s10585-010-9321-4 20229045

[32] Singh B, Berry JA, Shoher A, Ayers GD, Wei C, Lucci A (2007) COX-2 involvement in breast cancer metastasis to bone. Oncogene 26: 3789–3796. 17213821 10.1038/sj.onc.1210154

