# Supplementary figures and images for: Important Role of FTO in the Survival of Rare Panresistant Triple-Negative Inflammatory Breast Cancer Cells Facing a Severe Metabolic Challenge
Source: PLoS One. 2016 Jul 8;11(7):e0159072. doi: 10.1371/journal.pone.0159072 (PMC4938613; doi:10.1371/journal.pone.0159072)

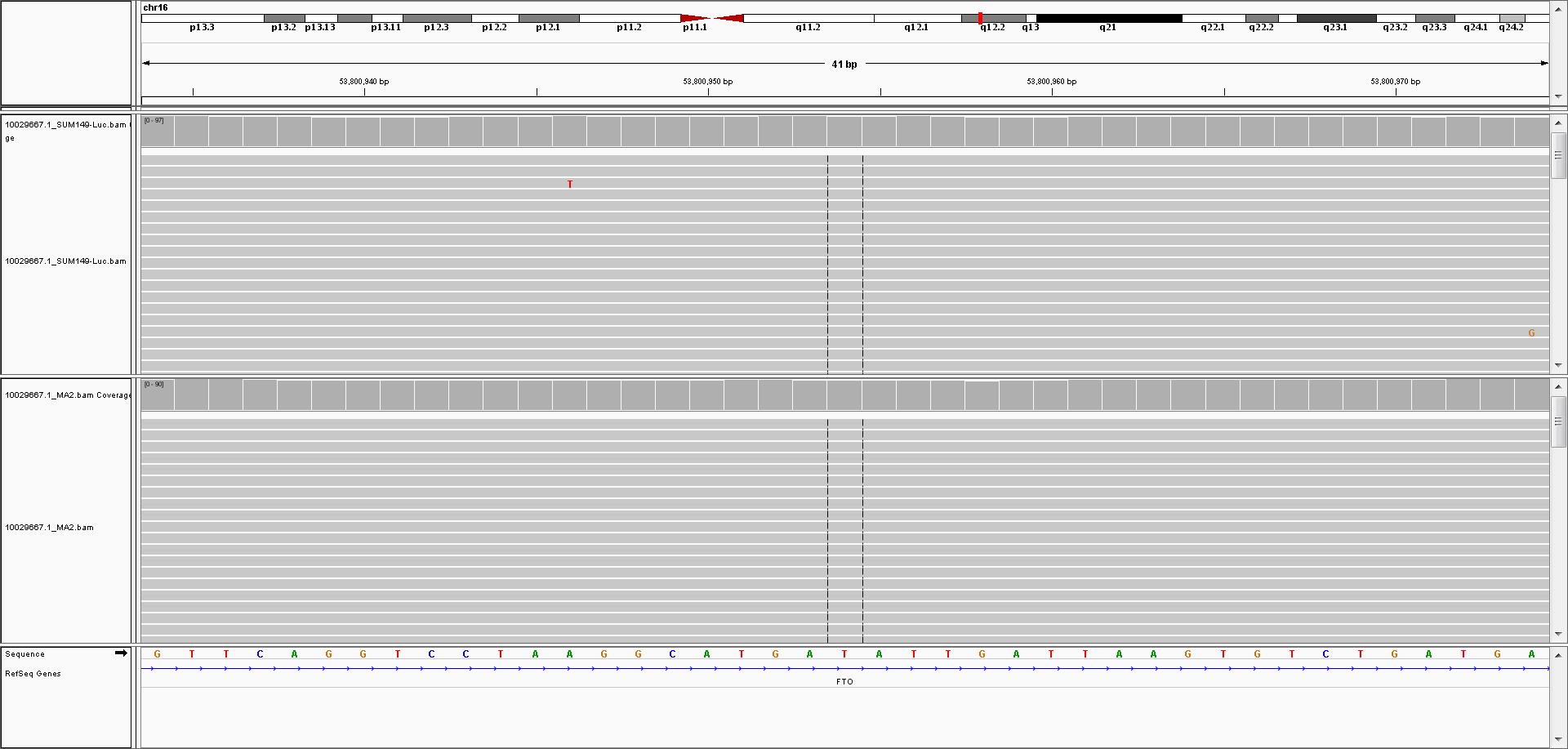

Supplement: S1 Fig — The whole genome DNA sequencing was performed with Illumina Genome Analyzer (Beckman Coulter Genomics) at an average 62.5X coverage. DNA sequences in binary alignment and map (BAM) format were aligned with the reference hg19 human genome in Integrative genomics Viewer (IGV version 2.3, Broad Institute, Boston, MA); SUM149-Luc (top), MA (middle), and hg19 reference (bottom). The T base included in the rs1421085 SNV, which renders it non-risk allele, is shown in the middle. Both SUM149-Luc and MA had only non-risk T allele. (JPEG) [file pone.0159072.s001.jpeg]

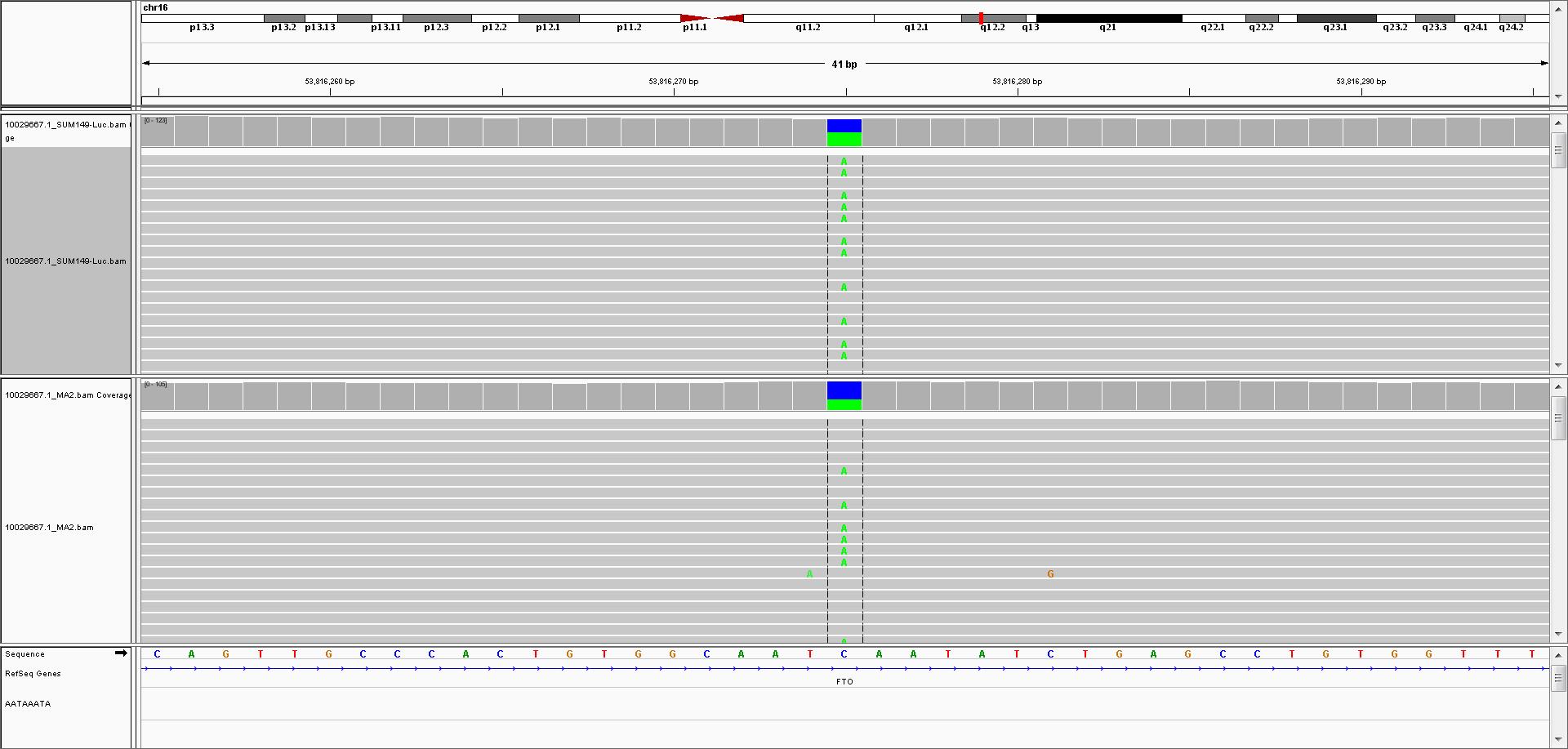

Supplement: S2 Fig — The DNA sequences were read and aligned with the reference hg19 human genome as in S1 Fig; SUM149-Luc (top), MA (middle), and hg19 reference (bottom). The C/A base included in the rs8050136 SNV, which renders it non-risk versus risk allele, is shown in the middle. Both SUM149-Luc and MA had both non-risk (C) and risk (A) alleles. However, the ratio of C:A was significantly different between the two cell lines: 49:51 in SUM149-luc versus 62:38 in MA. (JPEG) [file pone.0159072.s002.jpeg]

A

DMSO

MO-I-100  
2  $\mu$ MMO-I-500  
2  $\mu$ M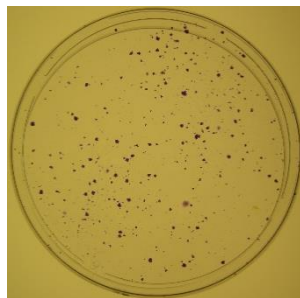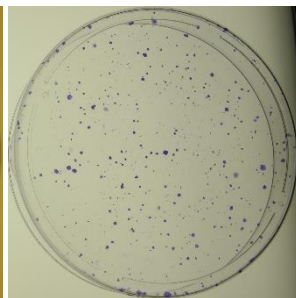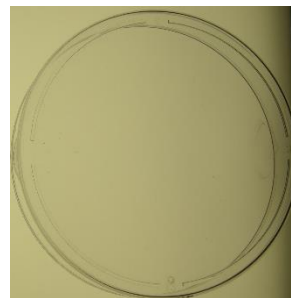

Colonies:

184

145

0

B

MO-I-500:  
0  $\mu$ M2.5  $\mu$ M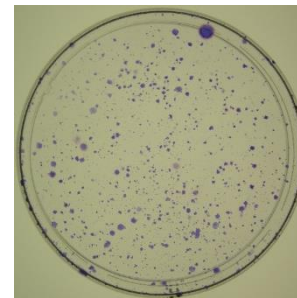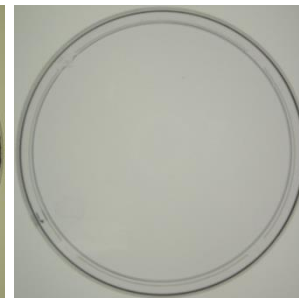

420

0

C

Dose: 1.5  $\mu$ M2.0  $\mu$ M

MO-I-100:

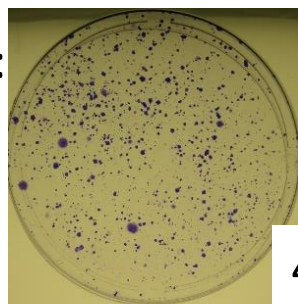

452

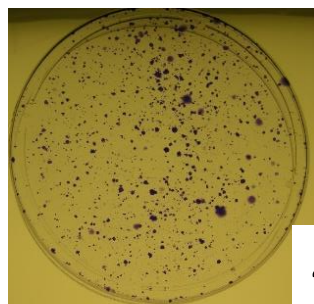

445

MO-I-500:

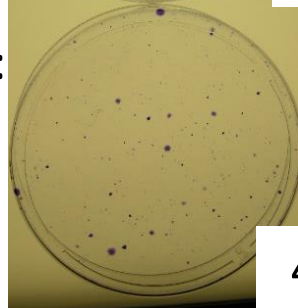

47

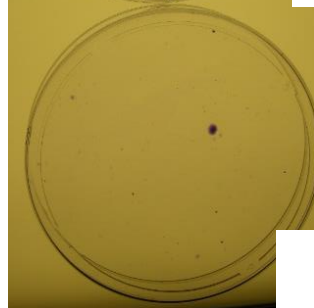

7

Supplement: S3 Fig — We plated SUM149-Luc cells with or without indicated doses of MO-I-500, MO-I-100, or DMSO solvent alone (0 dose), in a glutamine-free medium. We treated cells for different lengths of time, then washed off the drugs with phosphate-buffered saline, and allowed them to recover in glutamine-free medium without any drug before staining the colonies. Results from three separate experiments are shown: Panel A, treatment time 14 days and recovery time 8 days; panel B, treatment time 14 days and recovery time 20 days; panel C, treatment time 21 days and recovery time 1 day (this experiment is part of the experiment that is shown in Fig 2). The number of colonies is shown below the dishes (A and B) or on the lower right side of dishes (C). (PDF) [file pone.0159072.s003.pdf]

MO-I-500:

0  $\mu$ M

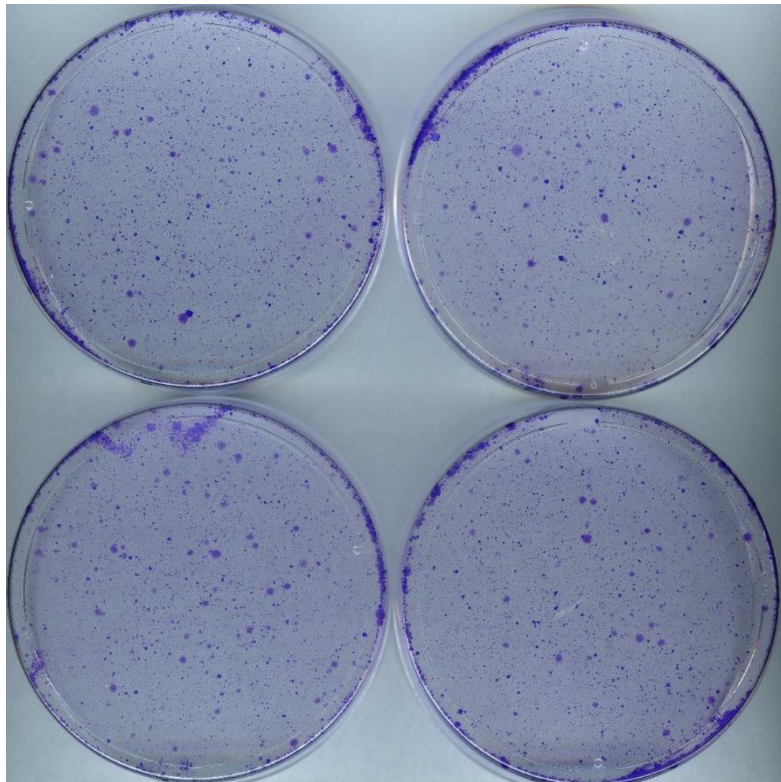

2  $\mu$ M

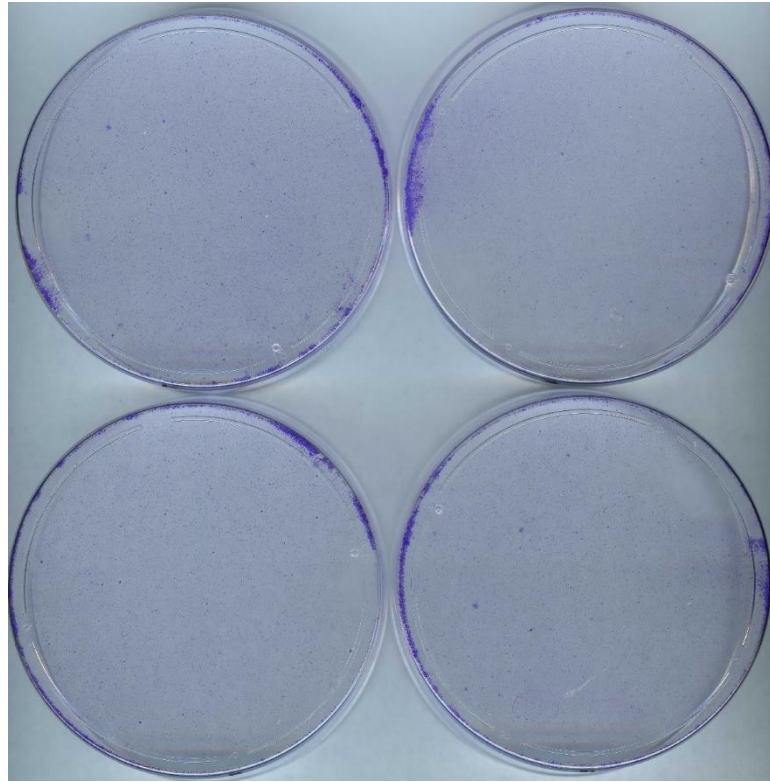

Colonies:

492 $\pm$ 80

12 $\pm$ 7

Supplement: S4 Fig — We plated SUM149-Luc cells in quadruplicate in 10 cm dishes with 2 μM MO-I-500 or DMSO solvent alone (0 dose) in a glutamine-free medium. We treated cells for 24 days and then stained the colonies with crystal violet. We obtained images in a scanner (Epson). Average number of colonies in treated and control groups along with standard deviation, as determined by the ImageJ software, is shown at the bottom. (PDF) [file pone.0159072.s004.pdf]

**A**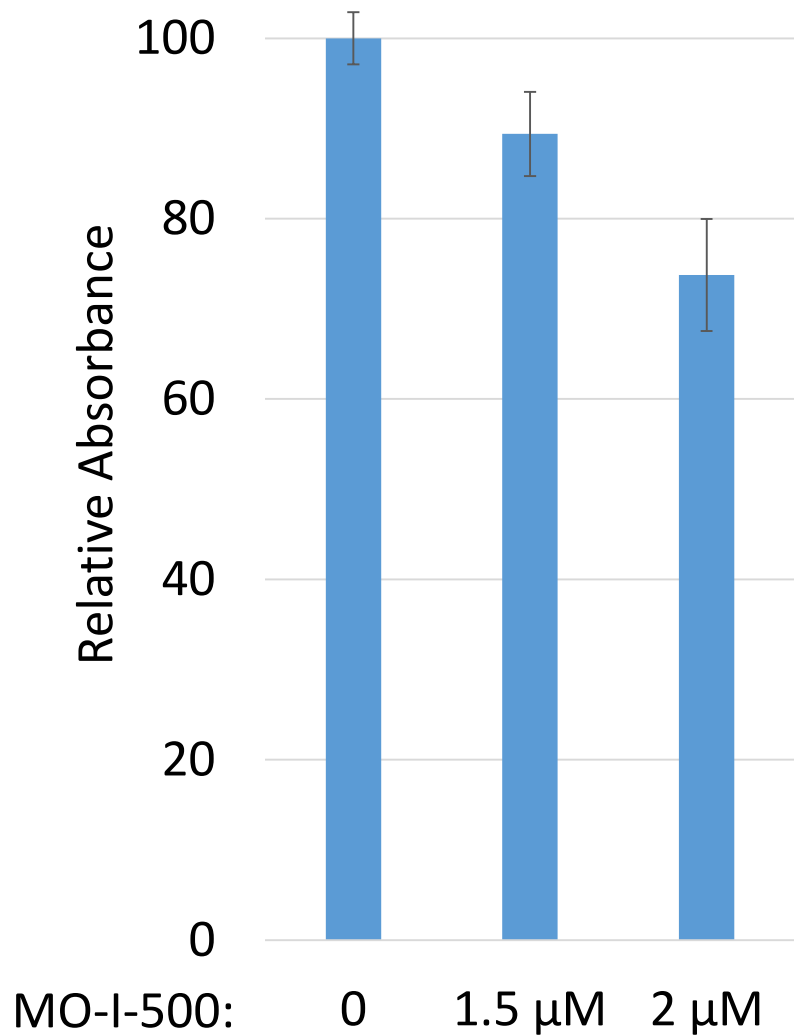**B**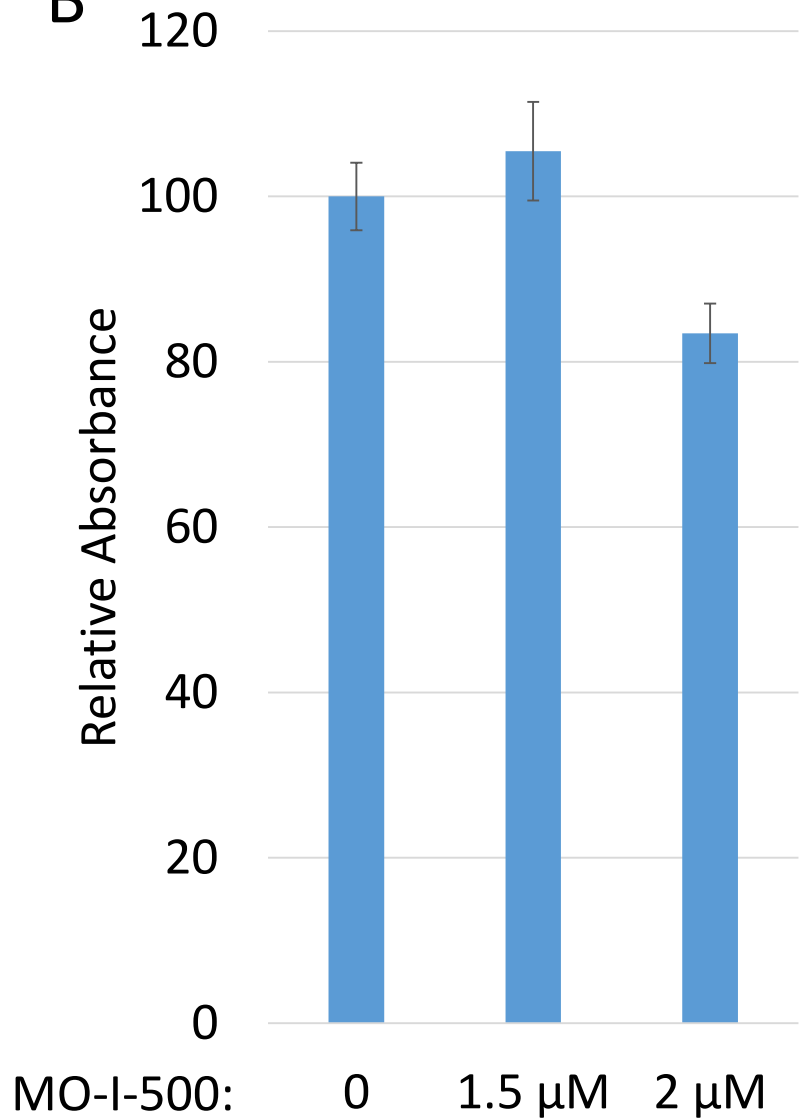

Supplement: S5 Fig — We incubated SUM149-Luc cells in 96-well plate along with MO-I-500 at the indicated concentrations in quadruplicate for 7 days, and then performed MTS cell proliferation assay. Controls were cells treated with DMSO alone (0 dose of MO-I-500). Relative average absorbance along with error bars representing standard deviation are shown. Panel A: cells growing in complete medium; panel B: cells growing in medium containing glutamine and dialyzed fetal bovine serum. The 100% absorbance values for DMSO-treated cells were 0.55 (panel A) and 0.35 (panel B). (PDF) [file pone.0159072.s005.pdf]

**A**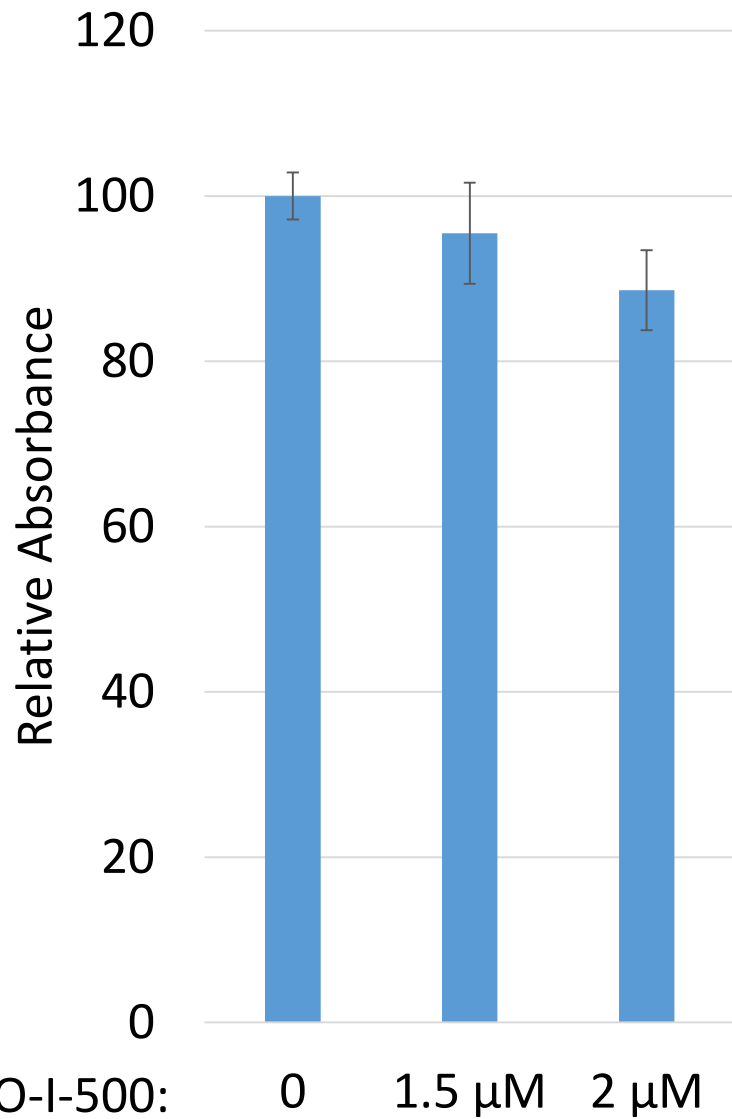**B**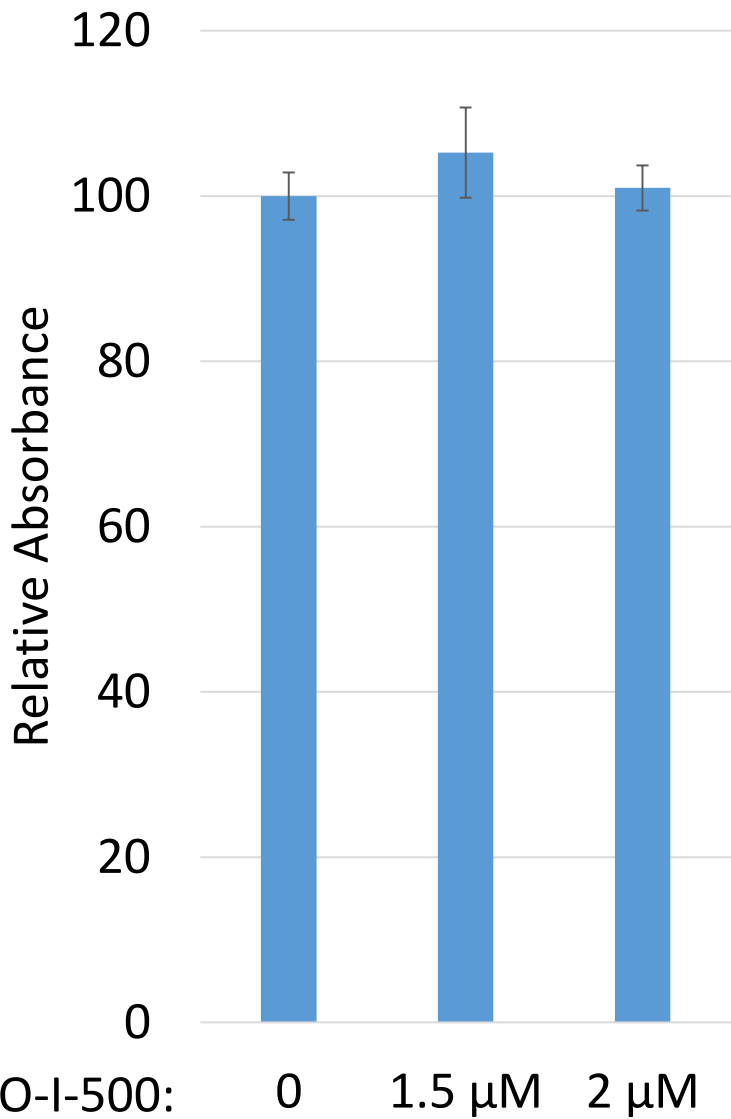

Supplement: S6 Fig — We incubated MA cells in 96-well plate along with MO-I-500 at the indicated concentrations in quadruplicate for 7 days, and then performed MTS cell proliferation assay. Controls were cells treated with DMSO alone (0 dose of MO-I-500). Relative average absorbance along with error bars representing standard deviation are shown. Panel A: cells growing in medium containing glutamine; panel B: cells growing in medium lacking glutamine. The 100% absorbance values for DMSO-treated cells are 0.39 (panel A) and 0.28 (panel B). We performed this experiment with the MA cells that were at passage 3 in glutamine-free medium after the initial selection. (PDF) [file pone.0159072.s006.pdf]
